# Supplementary material for: Consumption of protect and risk food groups for non-communicable diseases among women of reproductive age in rural areas of Morogoro, Tanzania
Source: PLoS One. 2025 Feb 25;20(2):e0318744. doi: 10.1371/journal.pone.0318744 (PMC11856286; doi:10.1371/journal.pone.0318744)
Supplement: Attachment — Submitted filename: PONE-D-24-02702_R2 +reviewer edits.pdf [file pone.0318744.s004.pdf]

PLOS ONE

Consumption of protective and risk food groups for non-communicable diseases among women of reproductive age in rural areas of Morogoro, Tanzania

--Manuscript Draft--

**Manuscript Number:**	PONE-D-24-02702R2
**Article Type:**	Research Article
**Full Title:**	Consumption of protective and risk food groups for non-communicable diseases among women of reproductive age in rural areas of Morogoro, Tanzania
**Short Title:**	Consumption of NCD-protect and NCD-risk food groups among women of reproductive age in rural Tanzania
**Corresponding Author:**	Akwilina Wendelin Mwanri, PhD<br>Sokoine University of Agriculture<br>Morogoro, TANZANIA, UNITED REPUBLIC OF
**Keywords:**	Diet quality, NCD-risk scores; NCD-protect scores; rural, women of reproductive age, Tanzania
**Abstract:**	Malnutrition has been persistently high in Tanzania, especially in the rural areas. Although some interventions have been implemented to reduce the rate of under nutrition, improvement is much slower than expected. Moreover, the prevalence of overweight and obesity among women of reproductive age has been on the increase. Changes in lifestyles specifically in eating habits may contribute to the observed increase trend in the prevalence of overweight and obesity. However, there are limited studies that have assessed diet quality for women. Hence, this study aimed to assess the consumption of protective and risk food groups for non-communicable diseases (NCD) in rural areas of the Morogoro region, Tanzania. The cross-sectional study involved 512 randomly selected women of reproductive age in Mvomero district. All mothers or caretakers with children of age 6 to 23 months who resided in the area for over three months were eligible to participate. A structured questionnaire and the Global Diet Quality Questionnaire for women were used to collect demographic and dietary intake data. A multivariate regression model was used to identify factors associated with consumption of diversified diets, NCD-protect, and NCD-risk food group scores. The results show that about 42% of the women had no formal education and about one in three women own a mobile phone. About 70% consumed vegetables while 33% consumed deep fried foods. Only 34% of the women met the minimum diet diversity (MDD) of five or more food groups. The mean NCD-risk score was 0.77±0.97 and the NCD-protect score was 2.8±1.4. Household income was positively associated with an increase in both NCD risk and protect-scores. Mobile phone ownership was found to be positively associated with NCD risk. Other factors such as age and agriculture were positively associated NCD protect while the number of children is negatively associated with NCD protect score. Rural communities should be educated on the importance of food diversification starting from production to consumption and the need for multiple sources of income to increase purchasing power of the household while considering proper food choices to avoid the risk of NCDs. The Ministry of agriculture and other stakeholders should support and promote production of bio-fortified crops and keeping small animals. The Ministry of Education should ensure that school children are aware of the NCD-risk foods and make available healthier food choices around the school environment.
**Order of Authors:**	Akwilina Wendelin Mwanri, PhD
	Fulgence Mishili, PhD
	Theresia Assenga, M.Sc.
	Rashid Suleiman, PhD
	Dismas Mwaseba, PhD
	Susan Nchimbi-Msolla, PhD
**Response to Reviewers:**	We appreciate comments from the Associate Editor. We have considered the

Powered by Editorial Manager® and ProduXion Manager® from Aries Systems Corporation

	comments and suggestions as guided. We hereby re-submit for consideration. Looking forward to your positive response.
Additional Information:	
**Question**	**Response**
**Financial Disclosure**<br><br>Enter a financial disclosure statement that describes the sources of funding for the work included in this submission. Review the submission guidelines for detailed requirements. View published research articles from *PLOS ONE* for specific examples.<br><br>This statement is required for submission and **will appear in the published article** if the submission is accepted. Please make sure it is accurate.<br><br>**Funded studies**<br>Enter a statement with the following details:<br>• Initials of the authors who received each award<br>• Grant numbers awarded to each author<br>• The full name of each funder<br>• URL of each funder website<br>• Did the sponsors or funders play any role in the study design, data collection and analysis, decision to publish, or preparation of the manuscript?<br><br>Did you receive funding for this work?	Yes
Please add funding details.<br>as follow-up to "**Financial Disclosure**<br><br>Enter a financial disclosure statement that describes the sources of funding for the work included in this submission. Review the submission guidelines for detailed requirements. View published research articles from *PLOS ONE* for specific examples.<br><br>This statement is required for submission and **will appear in the published article** if the submission is accepted. Please make sure it is accurate.	We declare that the research was funded by European Union's Horizon 2020 research and innovation programme under grant agreement [GA No 862802]. However, the funders had no role in study design, data collection and analysis, decision to publish, or preparation of the manuscript."

**Funded studies**<br>Enter a statement with the following details:<br>• Initials of the authors who received each award<br>• Grant numbers awarded to each author<br>• The full name of each funder<br>• URL of each funder website<br>• Did the sponsors or funders play any role in the study design, data collection and analysis, decision to publish, or preparation of the manuscript?<br><br>Did you receive funding for this work?"	
Please select the country of your main research funder (please select carefully as in some cases this is used in fee calculation).<br>as follow-up to "**Financial Disclosure**<br><br>Enter a financial disclosure statement that describes the sources of funding for the work included in this submission. Review the submission guidelines for detailed requirements. View published research articles from *PLOS ONE* for specific examples.<br><br>This statement is required for submission and **will appear in the published article** if the submission is accepted. Please make sure it is accurate.<br><br>**Funded studies**<br>Enter a statement with the following details:<br>• Initials of the authors who received each award<br>• Grant numbers awarded to each author<br>• The full name of each funder<br>• URL of each funder website<br>• Did the sponsors or funders play any role in the study design, data collection and analysis, decision to publish, or preparation of the manuscript?<br><br>Did you receive funding for this work?"	ITALY - IT

Powered by Editorial Manager® and ProduXion Manager® from Aries Systems Corporation

**Competing Interests**<br><br>Use the instructions below to enter a competing interest statement for this submission. On behalf of all authors, disclose any competing interests that could be perceived to bias this work—acknowledging all financial support and any other relevant financial or non-financial competing interests.<br><br>This statement is required for submission and **will appear in the published article** if the submission is accepted. Please make sure it is accurate and that any funding sources listed in your Funding Information later in the submission form are also declared in your Financial Disclosure statement.<br><br>View published research articles from *PLOS ONE* for specific examples.<br><br>**NO authors have competing interests**<br><br>Enter: *The authors have declared that no competing interests exist.*<br><br>**Authors with competing interests**<br><br>Enter competing interest details beginning with this statement:<br><br>*I have read the journal's policy and the authors of this manuscript have the following competing interests: [insert competing interests here]*<br><br>* typeset	The authors have declared that no competing interests exist
**Ethics Statement**<br><br>Enter an ethics statement for this submission. This statement is required if the study involved:<br><br>• Human participants<br>• Human specimens or tissue<br>• Vertebrate animals or cephalopods	The study was reviewed and approved by the Tanzania National Health Research Ethics Committee of the Institute for Medical Research (NIMR) with reference number NIMR/HQ/R.8a/VOL.IX/3605. Participants were informed on the study objectives, risks, benefits and procedures. Participation to the study was voluntary and all study participants who consented to participaate signed a written informed consent forms. The confidentiality of the data was ensured by using identification numbers rather than the names of the participants. Data was stored in secured laptop computers and only research team had the access.

• Vertebrate embryos or tissues<br>• Field research<br><br>Write "N/A" if the submission does not require an ethics statement.<br><br>General guidance is provided below. Consult the submission guidelines for detailed instructions. **Make sure that all information entered here is included in the Methods section of the manuscript.**	

Powered by Editorial Manager® and ProduXion Manager® from Aries Systems Corporation

**Format for specific study types**<br><br>**Human Subject Research (involving human participants and/or tissue)**<br>• Give the name of the institutional review board or ethics committee that approved the study<br>• Include the approval number and/or a statement indicating approval of this research<br>• Indicate the form of consent obtained (written/oral) or the reason that consent was not obtained (e.g. the data were analyzed anonymously)<br><br>**Animal Research (involving vertebrate animals, embryos or tissues)**<br>• Provide the name of the Institutional Animal Care and Use Committee (IACUC) or other relevant ethics board that reviewed the study protocol, and indicate whether they approved this research or granted a formal waiver of ethical approval<br>• Include an approval number if one was obtained<br>• If the study involved *non-human primates*, add *additional details* about animal welfare and steps taken to ameliorate suffering<br>• If anesthesia, euthanasia, or any kind of animal sacrifice is part of the study, include briefly which substances and/or methods were applied<br><br>**Field Research**<br><br>Include the following details if this study involves the collection of plant, animal, or other materials from a natural setting:<br>• Field permit number<br>• Name of the institution or relevant body that granted permission	
**Data Availability**<br><br>Authors are required to make all data underlying the findings described fully available, without restriction, and from the time of publication. PLOS allows rare exceptions to address legal and ethical concerns. See the PLOS Data Policy and FAQ for detailed information.	Yes - all data are fully available without restriction

A Data Availability Statement describing where the data can be found is required at submission. Your answers to this question constitute the Data Availability Statement and **will be published in the article**, if accepted.<br><br>**Important:** Stating 'data available on request from the author' is not sufficient. If your data are only available upon request, select 'No' for the first question and explain your exceptional situation in the text box.<br><br>Do the authors confirm that all data underlying the findings described in their manuscript are fully available without restriction?	
**Describe where the data may be found in full sentences. If you are copying our sample text, replace any instances of XXX with the appropriate details.**<br><br>• If the data are **held or will be held in a public repository**, include URLs, accession numbers or DOIs. If this information will only be available after acceptance, indicate this by ticking the box below. For example: *All XXX files are available from the XXX database (accession number(s) XXX, XXX.*).<br>• If the data are all contained **within the manuscript and/or Supporting Information files**, enter the following: *All relevant data are within the manuscript and its Supporting Information files.*<br>• If neither of these applies but you are able to provide **details of access elsewhere**, with or without limitations, please do so. For example:<br><br>*Data cannot be shared publicly because of [XXX]. Data are available from the XXX Institutional Data Access / Ethics Committee (contact via XXX) for researchers who meet the criteria for access to confidential data.*<br><br>*The data underlying the results presented in the study are available from (include the name of the third party*	Data will be available when the manuscript is accepted for publication

Powered by Editorial Manager® and ProduXion Manager® from Aries Systems Corporation

and contact information or URL).<br>• This text is appropriate if the data are owned by a third party and authors do not have permission to share the data.<br><br>* typeset	
Additional data availability information:	Tick here if the URLs/accession numbers/DOIs will be available only after acceptance of the manuscript for publication so that we can ensure their inclusion before publication.

Powered by Editorial Manager® and ProduXion Manager® from Aries Systems Corporation

Manuscript Click here to access/download;Manuscript;Manuscript.docx

Consumption of protect and risk food groups for non-communicable diseases among women of reproductive age in rural areas of Morogoro, Tanzania

Akwilina Wendelin Mwanri[1*], Fulgence Mishili[2¶], Theresia Assenga[2¶], Suleiman Rashid[3&], Dismas Mwaseba[4&], and Susan Nchimbi-Msolla[5&]

[1]Department of Human Nutrition and Consumer Sciences, Sokoine University of Agriculture, Morogoro, Tanzania

[2]Department of Agricultural Economics and Agribusiness, Sokoine University of Agriculture, Morogoro, Tanzania

[3]Department of Food Science and Agro-processing, Sokoine University of Agriculture, Morogoro, Tanzania

[4]Department of Agric. Extension and Community Development, Sokoine University of Agriculture, Morogoro, Tanzania

[5]Department of Crop Science and Horticulture, Sokoine University of Agriculture, Morogoro, Tanzania

*Corresponding author
Email: akwmwanri@sua.ac.tz **(AWM)**

[¶]These authors contributed equally to this work
[&]These authors also contributed equally to this work

Abstract

Malnutrition has been persistently high in Tanzania, especially in the rural areas. Although some interventions have been implemented to reduce the rate of undernutrition, improvement is much slower than expected. Moreover, the prevalence of overweight and obesity among women of reproductive age has been on the increase. Changes in lifestyles specifically in eating habits may contribute to the observed increase trend in the prevalence of overweight and obesity. However, there are limited studies that have assessed diet quality for women. Hence, this study aimed to assess the consumption of protective and risk food groups for non-communicable diseases (NCDs) in rural areas of the Morogoro region, Tanzania. The cross-sectional study involved 512 randomly selected women of reproductive age in Mvomero district. All mothers or caretakers with children of age 6 to 23 months who resided in the area for over three months were eligible to participate. A structure questionnaire was used to collect demographic data and the Global Diet Quality Questionnaire was used to collect dietary intake data for women. A multivariate regression model was used to identify factors associated with consumption of diversified diets, NCD-protect, and NCD-risk food group scores. The results show that about 42% of the women had no formal education and about one in three women own a mobile phone. About 70% consumed vegetables while 33% consumed deep fried foods. Only 34% of the women met the minimum diet diversity (MDD-W) of five or more food groups. The mean NCD-protect score was 2.8±1.4 and the NCD-risk score was 0.77±0.97. Household income was positively associated with an increase in both protect scores and NCD-risk. Mobile phone ownership was positively associated with NCD-risk. Other factors such as age and self-employment in agriculture were positively associated NCD-protect while the number of children was negatively associated with NCD protect score. Rural communities should be educated on the importance of food diversification starting from production to consumption and the need

for multiple sources of income to increase purchasing power of the household while considering proper food choices to avoid the risk of NCDs. The Ministry of Agriculture and other stakeholders should support and promote production of bio-fortified crops and keeping small animals. The Ministry of Education should ensure that school children are aware of the NCD-risk foods and make available healthier food choices around the school environment

Keywords: Diet quality, NCD-risk scores; NCD-protect scores; rural, women of reproductive age, Tanzania

Introduction

Despite the great progress made in improving food and nutrition security and reducing poverty over the past decades, undernutrition is still affecting billions of people worldwide [1]. Globally, about 170 million women are underweight; while three times as many women (610 million) are overweight [2]. Each year, about 20 million babies are born with low birth weight, which is closely associated with the eating behaviors and nutrition status of women before and during pregnancy [3]. Although undernutrition affects both urban and rural low-income households, those in the rural areas face more challenges than their urban counterparts. This is due to social isolation, intermittent drought, limited participation of women in economic activities, limited market access, inadequate access to health care, and other socioeconomic factors such as a lower education level and a high unemployment rate [4].

In Tanzania, undernutrition affects infants and children under the age of five, as well as women of childbearing age. [5]. Currently, about 7.3% of non-pregnant women aged 15–49 years are underweight [6]. According to the Tanzania Demographic Health Survey and Malaria Indicator Survey, the prevalence of chronic malnutrition [stunting] among children below five years in the

Morogoro region is high, at about 31% which is similar to the national average [6]. The prevalence of acute malnutrition is 4% which is above the national average and underweight is 10% [6]Studies show that undernutrition is particularly high among low-income rural Tanzanian households, mainly because they consume more carbohydrate-rich, staple-based diets that are low in minerals and vitamins [4,7]. Other factors contributing to malnutrition include frequent illness, low birth weight and inadequate childcare practices. In addition, only 46% of rural women in Tanzania meet their minimum dietary diversity requirement (i.e., consume at least 5 food groups out of 10 daily), and women's intake of protein, fruits, and vegetables is very low [4].

Rapid urbanization, along with the global economic crisis in developing countries, has put many urban and rural people at high risk of bad quality diets and malnutrition. There is a shift from consumption of traditional diets to more refined foods high in fat, sugar and salt even in the rural areas. In fact, many studies have shown that the population of women of reproductive age is at high risk of specific micronutrient deficiencies, which can affect their health [8,9,10,11]. Generally, the diets of Tanzanian women are less diversified and have a low intake of protein, fruits and vegetables [4]. Another study by Paul et al (2022) in Tanzania found that dietary quality was inversely associated with the risk of overweight and obesity [12]. Although there is an increased prevalence of overweight or obesity among women of reproductive age from 11% in 1991 to 36% in 2022 [6], most of the reported studies concentrated on assessing nutritional status and dietary diversity with limited information on unhealthy diets. Hence this study sought to assess the consumption of foods that increase the risk and those that are protective against diet-related NCDs among rural women of reproductive age.

Materials and methods

Description of the study area

A cross-sectional study was conducted in Mvomero District, Morogoro, specifically in the rural setting. Mvomero is one of the seven districts in Morogoro Region. The district is characterized by an average annual rainfall ranging between 487 and 1951 mm per year [13]. Based on the current population census, the total population for the district is 421,741, with 210,834 males and 210,907 females [14]. The district lies in three ecological zones: Miombo woodland, Highland, and mountains, as well as the Savanna River basin.

Some of the economic activities taking place in the area include agriculture, which employs about 75% of the district residents. Majority are small-scale farmers who produce various crops including maize, millet, cassava, sweet potatoes, beans, rice, sugar cane, cotton, and vegetables. Other activities done with the percentage of those involved in brackets are mining and quarrying employing (5%), trade and commerce (5%), and livestock keeping, fishing and hunting (3%). During the dry season, the majority of agricultural activities depend on the irrigation schemes established in the district which include Mkindo, Mlali, Mgeta, Ndole, Dihombo, Dakawa, and Komtonga irrigation schemes [13].

Data collection and sampling

Data were collected from November to December, 2022 from six villages located in Kinda Ward, namely Kinda, Magunga, Makuyu, Matale, Ndole, and Semwali. The sampling involved 518 randomly selected mothers with children of age between 6 and 23 months. The mothers were selected randomly from the village registry which had a list of all women with children of age 6 to 23 months. Six mothers/caretakers did not complete the study because they missed the dietary

intake data. All women who resided in the area for over three months were eligible to participate. Women known to be pregnant were excluded from the study because there could be pregnancy induced changes in food consumption. A structured, pre-tested questionnaire translated from English to Kiswahili was administered to collect data on socio-demographic characteristics, food availability, monthly income expenditure on food and decision-making in the household. In addition, the Global Diet Quality Questionnaire (GDQQ) – adapted for Tanzania for women was used to determine food group consumption, a ~~medium~~ dietary diversity score for women (MDD-W) and NCD-protect and NCD-risk scores based on 24-hr dietary recall [15]. The tools are available online, translated into Kiswahili and are free to be used by researchers [15]. The enumerator read the examples of the foods for each food group and marked one if any of the foods on the list within the food group were consumed or zero if none were consumed.

The 29 food groups from the GDQQ were aggregated to form nine NCD-protect and eight NCD-risk food groups. The NCD-Protect score (0-9) was based on food consumption from nine healthy food groups, namely whole grains, pulses, nuts and seeds, vitamin A-rich orange vegetables, dark green leafy vegetables, other vegetables, vitamin A-rich fruits, citrus, and other fruits [15]. A higher score indicates the inclusion of more health-promoting foods in the diet and correlates positively with meeting global dietary recommendations. Similarly, the NCD-risk score is a score with a range from 0 to 9. The score was based on the consumption of foods from eight food groups that are recommended to limit or avoid during the past day and night (one food group, processed meat, is double-weighted). The other food groups are soft drinks (sodas), baked / grain-based sweets, other sweets, unprocessed red meat, deep-fried food, fast food, instant noodles, and packaged ultra-processed salty snacks. A higher score indicates higher consumption of foods and drinks to avoid or limit.

Data analysis

STATA Statistical software was employed to analyze demographic factors, women's dietary diversity, and women's NCD- protect and risk scores, and the results were reported as descriptive. Some of the demographic factors used include monthly household income, a share of income spent on food, food shortage, mobile ownership, self-employment in agriculture, age, education level, sex of the household head, and respondents' number of children. Categorical variables were dichotomized and coded as one for those with the characteristics and zero for those without while discrete variables such as household income and number of children were used as continuous variables.

Econometric model: The study used a multivariate regression model to assess the determinants of women's NCD-protect and risk scores. The model is an extension of a simple linear regression model with the addition of more than one explanatory variable and outcome variables [16]. It can be used when the dependent variables are affected or influence by common independent variables. During analysis, two models with two dependent variables, that is, NCD-protect or risk scores, were analyzed with independent variables as indicated in equations 1 and 2. This study assumed that NCD-protect and risk scores can be influenced by common factors such as household monthly income, a share of income spent on food, food shortage, mobile ownership, employment, self-employed in agriculture (agricultural), age, education level, sex of the household head, and respondents' number of children. The variable of household income was transformed into a log to ensure data are normally distributed.

$$\text{NCD Protectscores} = \beta_0 + B_1\text{Monthly Household Income} + \beta_2\text{Mother's age is above35Years} + \beta_3\text{Mother's age is between 26 to 35 Years} + \beta_4\text{Share of food expenditure is about 50\%} + \beta_5\text{Share food expenditure is above 50\%} + \beta_6\text{Total number of Children} + \beta_7\text{Secondary Education and Above} + \beta_8\text{Primary Education} + \beta_9\text{ Agricultural } + \beta_{10}\text{Mobile Access} + \beta_{11}\text{Sex of Household Head is male} + \varepsilon \ldots\ldots\ldots..\ldots\ldots \text{(Equation1)}$$

$$\text{NCD Risk} = \beta_0 + B_1\text{Monthly Household Income} + \beta_2\text{Mother's age is above 35Years} + \beta_3\text{Mother's age is between 26 to 35 Years} + \beta_4\text{Share of food expenditure is about 50\%} + \beta_5\text{Share of food expenditure is above 50\%} + \beta_6\text{Total number of Children} + \beta_7\text{Secondary Education and Above} + \beta_8\text{Primary Education} + \beta_9\text{s} + \beta_{10}\text{Mobile Access} + \beta_{11}\text{Sex of Household Head} + \varepsilon \ldots\ldots\ldots..\ldots\ldots \text{(Equation 2)}$$

Minimum dietary diversity for women

Ten food groups that includes, "grains (white roots and tubers, and plantains), pulses (beans, peas and lentils), nuts and seeds, dairy, meat (poultry and fish), eggs, dark green leafy vegetables, other vitamin A-rich fruits and vegetables, other vegetables and fruits" were used to determine the MDD-W [17,18].. Each food was assigned a weight of 1 if consumed and 0 otherwise to give a total of 10 if all food groups were consumed in the 24 hours preceding the survey [15, 17, 18].

Women who consumed five or more food groups were regarded as attaining a minimum dietary diversity (1), while those who consumed less than five food groups were considered otherwise (0). A binary logit regression model with a dependent variable of women's minimum dietary diversity was used to assess the influence of social-demographic factors on women's dietary diversity.

Ethical consideration

Permission to conduct the study was sought from Sokoine University of Agriculture and the regional, district, ward, and village authorities. The study was approved and ethical clearance obtained from National Health Research Ethics Committee of the Tanzania National Institute for Medical Research (NIMR) with reference number NIMR/HQ/R.8a/VOL.IX/3605. Participants were informed about the objectives of the study, the procedure, benefits, and risks. Written consent was obtained from those who agreed to participate. Participation was voluntary and each participant was free to decide otherwise at any time. All participants were identified by code numbers; data was kept in a secured computer and were accessible to the research team only.

Results

The mean age of the respondents was 26.4 (SD7.3), ranging from 15 to 49 years. More than 50% of the respondents were of age below 25 years. About 42% of the respondents did not have any formal education, and one in three owned a mobile phone. About 45% spent more than half of their income on food (Table 1).

Table 1: Summary of descriptive analysis for demographic and other factors (N = 512)

**Variables**		**n (%)**
	15 to 25 years	284(55)
Age class	26 to 35years	162(32)
	Above 35 years	66(13)
	Less than 50	142(28)
Share of income on food	50% Share	141(28)
	Above 50	229(45)
	No	337(66)
Mobile ownership	Yes	175(34)
	No formal education	215(42)
Education level	Primary education	265(52)
	Secondary and above	32(6)
Agricultural	No	47(9)
	Yes	465(91)
Gender of the household head	Male household head	464(91)
	Female household head	48(9)

Descriptive results for numeric variables

The mean monthly household income was 112,569TZS (47USD) ranging from 3000TZS (1.3USD) to 1,550,000 TZS (633USD)**.** The average number of children per household was about three children, ranging from one to eleven children (Table 2).

Table 2: Descriptive results for numeric variables

Variable	Observation	Mean	Std. Dev.	Min	Max
Household income (TZS)	512	112,569 (47USD)	138,835 (57USD)	3000TZ (1.3USD)	1,550,000TZS (633USD)
Number of children	512	3	2	1	11

Consumption of NCD-protect and NCD-risk food groups (N = 512)

Table 3 shows consumption of food groups that are considered healthy or unhealthy for women. Regarding healthy foods, vegetables and pulses were commonly consumed by a large proportion of women (72% and 44%), respectively. However, only 16% of women consumed whole grains. Among the unhealthy foods, relatively high proportion of women consumed deep fried foods (33%), and baked/grain based sweet foods (15%) ~~were consumed as unhealthy food~~.

Table 3: Consumption of NCD-protect and NCD-risk food groups (N = 512)

NCD Protect food groups	n (%)	NCD Risk food groups	n (%)
Whole grains	82(16)	Sugar sweetened beverages	28(6)
Pulse	226(44)	Baked/grains based sweets	77(15)
Nuts and seeds	54(11)	Other sweet	45(9)
Vitamin A rich orange vegetables	16(3)	Processed meat	0(0)
Dark green leafy vegetables	361(71)	Unprocessed red meat	38(7)
Other vegetables	370(72)	Deep-fried foods	168(33)
Vitamin A rich fruits	126(25)	Fast foods and instant noodles	2(0.03)
Citrus	17(3)	Processed packed salty snacks	2(0.03)
Other fruits	164(32)		

Descriptive analysis for women NCD Protect and Risk scores

The mean NCD-protect score was 2.8±1.4, with a range from 0 to 8 food groups while the NCD-risk score around 0.7±0.9 with a range of 0 to 5 food groups (Table 4).

The mean NCD protect score was higher among women who spent about half of their income on food (mean 3.0 ±1.5) and higher among women with a single child (0.9±1.0) (Table 4).

Table 4: Descriptive analysis for women NCD protect (mean+/-SD) and NCD-risk (mean+/-SD) Scores

**Variables**		**Observations**	***NCD-Protect**	***NCD-Risk**
	15 to 25 years	282	2.8±1.4	0.8±0.96
Age class	26 to 35 years	163	2.7±1.5	0.7±1.1
	Above 35 years	67	2.6±1.4	0.5±0.8
	1 child	161	3.0±1.5	0.9±1.0
Number of children	2 to 3 children	208	2.7±1.4	0.7±1.0
	Above 3 children	143	2.6±1.4	0.6±1.0
	Less than 50%	142	2.9±1.6	0.8±1.0
Share of income on food	About 50%	141	3.0±1.5	0.76±0.9
	Above 50%	229	2.6±1.3	0.6±0.98
	No	337	2.7±1.5	0.6±0.9
Mobile ownership	Yes	175	2.9±1.4	0.9±1.1
	No formal education	215	2.7±1.4	0.7±1.0
Education level	Primary education	266	2.8±1.5	0.7±1.0
	Secondary and above	31	2.7±1.3	0.9±0.9
agricultural	No	47	2.4±1.3	0.7±0.8
	Yes	465	2.8±1.5	0.7±1.0

Gender of the household head	Male household head	463	2.8±1.4	0.7±1.0
	Female household head	49	2.4±1.4	0.7±0.8
Total			2.8± 1.4	0.71±0.97

*Value in the table mean ± standard deviation

Influence of demographic factors to women NCD- protect and NCD-risk scores.

The multivariate regression model shows that there was a significant association between the NCD-protect score and household income, age (above 35 years), number of children in the household, agricultural employment, and sex of the household head. There was a significant positive association between household income and mobile phone ownership with NCD-risk score (Table 5).

Table 5: Influence of demographic factors to women NCD- Protect and risk scores

	NCD Protect			NCD Risk		
**Variables**	**Coefficients**	**S.E**	**P>t**	**Coefficients**	**S.E**	**P>t**
Household income (Log)	0.299	0.059	0.000***	0.254	0.040	0.000***
Above 35 years	0.784	0.316	0.013**	-0.043	0.213	0.841
Age 26 to35 years	0.293	0.184	0.112	0.012	0.124	0.923
About 50% share of income on food	0.168	0.171	0.326	0.052	0.115	0.65
Above 50% share of income on food	-0.248	0.152	0.103	-0.142	0.102	0.165
Number of children	-0.205	0.057	0.000***	-0.041	0.039	0.285
Secondary education and above	-0.198	0.297	0.506	0.043	0.200	0.83
Primary Education	0.063	0.136	0.646	-0.142	0.092	0.123
Agricultural	0.458	0.226	0.043**	0.108	0.152	0.48
Mobile Ownership	0.084	0.141	0.55	0.211	0.095	0.027**
Sex of Household head	0.369	0.222	0.097*	0.016	0.150	0.916

Constant	-0.901	0.724	0.214	-2.044	0.488	0.000

Equation	RMSE	R-sq.	F
NCD Protect	1.40	0.10	4.90**
NCD Risk	0.94	0.11	5.35**

*** Significant at 1%, ** Significant at 5% and * Significant at 10%.

Minimum dietary diversity for women and its determinants

Tables 6 and 7 show that only one-third of the women (34%, n=174) attained the MDD-W. Household income, age of the mother, number of children, ownership of a mobile phone and sex of the household head were associated with the MDD-W

Table 6): Minimum dietary diversity for women

Women dietary diversity	N	%	Mean	S.D	Min	Max
MDD-W<5	338	66	3	1	1	4
MDD-W≥5	174	34	6	1	5	10

Table 7: Determinants of women minimum dietary diversity

Independent Variables	Odds Ratio	Robust S.E	P>z	95% Conf. Interval	
Household income (log)	1.6847	0.1615	0.000***	1.3961	2.0330
Above 35 years	3.8429	2.0441	0.011**	1.3549	10.8999
Age 26 to 35 years	1.7504	0.5243	0.062	0.9732	3.1485
About 50% share of income on food share	1.0886	0.2956	0.755	0.6393	1.8535
Above 50% share of income on food	0.6727	0.1643	0.105	0.4168	1.0858
Number of children	0.7084	0.0710	0.001***	0.5820	0.8623
Secondary education and Above	0.9968	0.4552	0.994	0.4073	2.4395
Primary Education	1.1997	0.2652	0.41	0.7779	1.8503
Agricultural	1.8369	0.6798	0.1	0.8894	3.7939
Mobile Ownership	1.7683	0.3981	0.011**	1.1374	2.7490
Sex of household head	2.6168	1.0126	0.013**	1.2257	5.5866
Constant	0.0006	0.0007	0.000	0.0001	0.0057

*** Significant at 1%, ** Significant at 5% and * Significant at 10%.

Discussion

This study aimed to assess the consumption of food groups that increase the risk and those that are protective against diet related NCDs among women of reproductive age in rural areas of Morogoro, Tanzania. There are limited number of studies that used GDQQ to estimate minimum dietary diversity, NCD-protect and NCD-risk scores among women of reproductive age in Tanzania [based on 24-hr dietary recall] [15]. The findings of this study indicate that the mean NCD-protect and NCD-risk scores for women were low, but the mean NCD-protect score was higher than that of the risk score, implying that rural women are more protected from NCDs as far as food intake is concerned. The lower consumption of NCD-risk food groups observed in this study can possibly be due to the fact that women living in rural areas have limited access and low purchasing power and hence depend mainly on what they produce. This finding is consistent with a review done by Akumu et al [19], which shows that, the diet in Uganda is mainly composed of starch staples, especially roots, tubers, bananas, and legumes which are healthy foods. A study conducted in Peru among women of reproductive age reported a higher prevalence of consumption of unhealthy foods, such as sugar-sweetened beverages, consumption ~~in~~ by 97% [20].

A high proportion of women consumed vegetables and pulses in the healthy food groups. The observed practice is not surprising because the villages involved in the study produce pulses and plenty of vegetables in the river valleys. This finding is in line with the one reported in a study conducted in rural areas of Kenya, which revealed that vegetables and pulses were mostly consumed by women in rural areas [21]. However, contrasting results have been shown in other studies with a shortfall of fruits and vegetable intake in the diet of women of reproductive age [22,23,24]. Nonetheless, the findings of this study are inconsistent with several other studies that reported that the most commonly consumed food groups by women of reproductive age in rural areas were staple foods such as grains or cereal, roots and tubers [25,23,26]. These results can

possibly be explained by seasonality, socioeconomic variation among the study groups, as well as the availability of these food groups in households where vegetables are commonly produced and accessed from the market at a low price. However, the reported consumption was qualitative hence not necessarily imply that the studied women meet recommended amount of vegetable intake.

Deep-fried foods and baked/grain-based sweets were the most commonly consumed unhealthy foods. The high consumption of deep-fried foods shows changes in food preparation methods from common boiling or steaming to deep frying due to the availability and access of cooking oil, and shifts in dietary preferences, taste/palatability and lifestyles. Other studies conducted in Tanzania reported similar findings that about 21% of the rural households consume ultra-processed foods and foods away from home which are high in fat, sugar and salt [27,28].

From regression analysis, household income was positively associated with both NCD-protect and NCD-risk scores. This means households with more income can access more diversified diets, with some being in the healthy and others in the unhealthy groups. Higher-income households often have more disposable income, making it easier for them to afford a wide variety of foods, including healthier and unhealthy options. Nevertheless, lower-income households often face greater challenges in accessing and affording healthier food options, which can lead to consumption of monotonous, less diversified foods based on what they produce or what they can afford. More food access and availability due to high income may not necessarily lead to healthier option. It was reported that nutritious foods eaten at home in Tanzania has not improved with rising of income [27]. A study done in Tanzania using Household Budget Survey data support the current findings that lower income households purchase fewer healthful foods compared with higher income households [29]. Another study conducted in the in Sub-Saharan Africa reported similar

findings; lower food expenditure is likely to be a key contributor to less healthy food choices among lower socioeconomic groups [30].

In this study, being older than 35 years was associated with greater consumption of NCD-protect food groups than their younger counterparts. With age, people often make lifestyle changes to prioritize health and well-being, including dietary modifications, as they develop more established and health-conscious dietary habits, which can include a greater focus on consuming NCD-protect foods [31]. It could also be explained by their being more exposed to nutrition education compared to the younger women. Another reason might be health conditions, as older individuals may have pre-existing health conditions that necessitate dietary changes, encouraging the consumption of NCD-protect foods. The finding of the current study is in line with a study conducted in Australia and Nepal, which found that being older than 35 years had higher component scores in limiting consumption of NCD-risk food groups, such as limiting consumption of saturated fat and consuming low-fat milk [32,33].

Furthermore, there were a significant negative association between the NCD-protect score and the number of children in the households. This means households with fewer children had higher NCD-protect scores compared to those with more children. This implies that households with few children are more likely to diversify their diet. These findings are supported by results from a systematic review and meta-analysis conducted in Ethiopia, which reported that, pregnant women living in larger families were more likely to consume less diversified diets compared to those living in smaller families [34]. This can possibly be explained by the fact that families with more children may have to allocate their resources, including food, among a greater number of individuals, which could affect the quality and diversity of their diet and strain the economic resources of a household, limiting their ability to purchase a variety of NCD-protect foods. It might as well be related to time

constraints that make it more challenging to prepare and consume healthier meals, potentially leading to reliance on more convenient but less nutritious options. Despite this, smaller families might have more time to plan and prepare nutritious meals, leading to increased consumption of healthy foods.

Having a mobile phone was significantly associated with NCD-risk scores. Mobile phones enable exposure to digital marketing and advertising, potentially influencing food choices and consumption patterns [35]. Additionally, women who own mobile phones may be more influenced by friends or family members to consume unhealthy foods. They could also be doing business outside the home, hence purchasing unhealthy foods sold around by the food vendors, which are mainly french fries, African doughnuts, and fried bananas or cassava. Findings of this study are inconsistent with those reported in a study conducted in Ethiopia and Uganda, which revealed that owning a mobile phone was significantly associated with a wide intake of a variety of food groups due to access to nutrition and health information [36,37].

Factors associated with minimum dietary diversity for women of reproductive age

Regression analysis indicated that being older was associated with MDD-W. This can possibly be explained by the fact that older individuals often have more diverse food preferences and culinary experiences accumulated over time [38]. Economic stability and access to a wider range of foods may also increase with age, allowing older women to have more opportunities to diversify their diets. Findings of this study are in line with the studies conducted in Lesotho, Kenya and India which reported older individuals having a higher minimum dietary diversity compared

to their younger counterparts [39,40]. Additionally, as people age, they may develop a greater awareness of the importance of a varied diet for overall health and well-being. It is also possible that older people may have more income from the economic activities they have established over the years than the younger ones which enable then to diversify foods more. They may also receive some support from their children who are working outside the village.

Higher-income households can contribute to greater dietary diversity due to increased access to a variety of nutritious foods. High-income households often have the financial means to purchase a wider range of fruits, vegetables, proteins, and other essential nutrients, promoting a more diverse and balanced diet [41]. Additionally, economic stability reduces food insecurity, allowing for consistent access to different food groups, which is crucial for maintaining nutrition status. The findings of this study are supported by a study in Addis Ababa, Ethiopia [42] and Lesotho [39].

Having a mobile phone in rural areas among women could be associated with higher dietary diversity due to improved access to information about nutrition, agricultural practices, and market prices. Mobile phones enable women to receive timely updates on farming techniques, access weather forecasts, and connect with markets, allowing them to make informed decisions about crop choices and food purchases [43]. The findings of this study are in line with the study conducted in Jerdu, Ethiopia by [36] which found that women who own mobile phones were more likely to have ~~a~~ minimum dietary diversity ~~score~~ than their counterparts. Moreover, mobile phones can facilitate communication within communities, creating opportunities for knowledge-sharing related to diverse and nutritious food options among women.

Minimum dietary diversity score for women was higher in male headed households than female headed ones. This can possibly be explained by male heads of households may have greater access to resources, which can facilitate the purchase of healthier food options and wide variety of foods

[1]. It might as well be related to the demand of household chores which may limit the time and energy to plan and prepare a wide variety of foods in female headed households. Findings of this study are supported by the study done in Tanzania and Burkina Faso, which found that MDD-W was lower in female headed households than their counterparts [1,44].

In addition, number of children in the households was inversely associated with minimum dietary diversity. This finding can possibly be explained by the fact that a smaller number of children in households may lead to greater dietary diversity, there being more resources available per child, allowing for a wider range of food options. Additionally, smaller families might be more flexible in adapting to diverse food preferences and accommodating a wider range of foods. This contrasts with larger families, where resource constraints and time limitations might result in a more limited variety of foods. Studies done in Nakuru county Kenya and Lesotho reported a similar finding that household size can also influence adequate dietary diversity among women of reproductive age [38,39].

Conclusion

Generally, the mean NCD-protect score was higher than the NCD-risk score, implying that rural women are more protected from NCDs as far as food intake is concerned. Although the mean NCD-risk was low, about one out of three women consumed deep-fried foods in the day preceding the survey. Household income, mobile ownership, age, sex of the household head, and respondents' number of children were strongly associated with NCD-protect and risk scores. Only one in three women met the recommended MDD-W implying that majority may have inadequate micronutrient intake. Therefore, rural communities should be supported to diversify food

production and sources of income to increase purchasing power while considering proper food choices to increase micronutrients intake and avoid the risk for NCDs. The Ministry of Agriculture and other stakeholders should support and promote production of bio fortified crops and keeping small animals. The Ministry of Education should ensure that school children are aware of the NCD-risk foods and make available healthier foods choices around the school environment

It is important to note that the study was conducted in one region (Morogoro) out of 31 regions and on one district (Mvomero) out of the eight districts of Morogoro region, Tanzania; hence the data may not be generalized to other regions due to variations across the regions.

Acknowledgements

We acknowledge the study participants for accepting to participate in the study. We appreciate the support from the field assistants and we thank the regional, district and Sokoine University of Agriculture authorities for providing support to carry out the study**.** We acknowledge support from FoodLAND project members who contributed to this study in one way or the other.

References

1. Ochieng J, Afari-Sefa V, Lukumay PJ, Dubois T. Determinants of dietary diversity and the potential role of men in improving household nutrition in Tanzania. PLoS One. 2017;12[12]:1–18.
2. United Nations Children's Fund. UNICEF Programming Guidance. Prevention of malnutrition in women before and during pregnancy and while breastfeeding [Internet]. Caring for Children Born Small for Gestational Age. New York; 2021. Available from:

https://www.unicef.org/media/115361/file/Maternal
3. United Nations Children's Fund and World Health Organization. Low Birthweight Estimates: Levels and trends 2000-2015. Vol. 7, The Lancet Global Health. Geneva; 2019.
4. Madzorera I, Blakstad MM, Bellows AL, Canavan CR, Mosha D, Bromage S, et al. Food Crop Diversity, Women's Income-Earning Activities, and Distance to Markets in Relation to Maternal Dietary Quality in Tanzania. Journal of Nutrition [Internet]. 2021;151[1]:186–96. Available from: https://doi.org/10.1093/jn/nxaa329
5. Ministry of Health, Community Development, Gender, Elderly and Children [MoHCDGEC] [Tanzania Mainland], Ministry of Health [MoH] [Zanzibar], Tanzania Food and Nutrition Centre [TFNC], National Bureau of Statistics [NBS], Office of the Chief Government Statistician and U. Tanzania National Nutrition Survey using SMART Methodology [TNNS] 2018. Dar es Salaam, Tanzania;2019.
6. Ministry of Health [MoH] [Tanzania Mainland], Ministry of Health [MoH] [Zanzibar], National Bureau of Statistics [NBS], Office of the Chief Government Statistician [OCGS], and ICF. Tanzania Demographic and Health Survey and Malaria Indicator Survey 2022 Key Indicators Report. 2023. Availablefrom:https://www.nbs.go.tz/nbs/takwimu/dhs/Tanzania_DHS-MIS_2022_Final_Report.pdf
7. Huang M, Sudfeld C, Ismail A, Vuai S, Ntwenya J, Mwanyika-Sando M, et al. Maternal Dietary Diversity and Growth of Children Under 24 Months of Age in Rural Dodoma, Tanzania. Food Nutrition Bulletin. 2018;39[2]:219–30.
8. Brazier AKM, Lowe NM, Zaman M, Shahzad B, Ohly H, McArdle HJ, et al. Micronutrient status and dietary diversity of women of reproductive age in rural Pakistan. Nutrients. 2020;12[11]:1–15.
9. Chaudhary SR, Govil S, Lala MK, Yagnik HB. Infant and young child feeding index and its association with nutritional status: A cross-sectional study of urban slums of Ahmedabad. Journal of Family and Community Medicine. 2018;25[2]:88–94.
10. Agada M, Igbokwe E. Dietary Diversity of Rural Households in North Central Nigeria. European

Journal of Nutrition and Food Safety. 2015;5[3]:150–5.

11. Nupo SS, Oguntona CRB, Onabanjo OO, Fakoya EO. Dietary diversity scores and nutritional status of women in two seasons in rural areas of Ogun State, Nigeria. Nutrition Food Science. 2013;43[1]:60–7.

12. Paulo HA, Mosha D, Mwanyika-Sando M, Mboya IB, Madzorera I, Killewo J, et al. Role of dietary quality and diversity on overweight and obesity among women of reproductive age in Tanzania. PLoS One [Internet]. 2022;17[4 April]:1–13. Available from: http://dx.doi.org/10.1371/journal.pone.0266344

13. United Republic of Tanzania. Mvomero district investment profile. 2017. Available from: https://mvomerodc.go.tz/storage/app/uploads/public/5bb/da6/b9b/5bbda6b9b955b438674778.pdf

14. United Republic of Tanzania. Administrative Units Population Distribution Report. National Population and House Census of Tanzania. National Bureau of Statistics, Dar es Salaam, Tanzania.2022.Availablefrom:https://www.nbs.go.tz/nbs/takwimu/Census2022/Administrative_units_Population_Distribution_Report_Tanzania_volume1a.pdf

15. Global Diet Quality Project. DQQ for Tanzania. Accessed at dietquality.org https://www.dietquality.org/tools and https://www.dietquality.org/countries/tza

16. Hidalgo B, Goodman M. Multivariate or multivariable regression? American Journal of Public Health. 2013;103[1]:39–40.

17. Martin-Prevel Y, Arimond M, Allemand P, Wiesmann D, Ballard TJ, Deitchler M, et al. Development of a dichotomous indicator for population-level assessment of dietary diversity in women of reproductive age. Current Development Nutrition. 2018;1[12].

18. Food and Agriculture Organization, 360 FHI. Minimum Dietary Diversity for Women: A Guide for Measurement [Internet]. 2016. 176 p. Available from: http://www.fao.org/3/a-i5486e.pdf

19. Akumu G, Ogenrwoth B, Mugisha J, Muyonga JH. Dietary Patterns in Uganda and Their Influencing Factors: a Critical Review. African Journal of Food, Agriculture, Nutrition and Development. 2023;23[2]:22328–53.

20. Pradeilles R, Pareja R, Creed-Kanashiro HM, Griffiths PL, Holdsworth M, Verdezoto N, et al. Diet and food insecurity among mothers, infants, and young children in Peru before and during COVID-19: A panel survey. Maternal and Child Nutrition. 2022;18[3].

21. Kishino M, Hirose M, Hida A, Tada Y, Ishikawa-Takata K, Hara K, et al. Characteristics of Dietary Intake in Relation to the Consumption of Home-Produced Foods among Farm Women in Two Rural Areas of Kenya: A Preliminary Study. Dietetics. 2022;1[3]:242–54.

22. Otunchieva A, Smanalieva J, Ploeger A. Dietary Quality of Women of Reproductive Age in Low-Income Settings: A Cross-Sectional Study in Kyrgyzstan. Nutrients. 2022;14[2].

23. Keno S, Bikila H, Shibiru T, Etafa W. Dietary diversity and associated factors among children aged 6 to 23 months in Chelia District, Ethiopia. BMC Pediatrics. 2021;21[1]:1–8.

24. Naicker A, Venter CS, MacIntyre UE, Ellis S. Dietary quality and patterns and non-communicable disease risk of an Indian community in KwaZulu-Natal, South Africa. Journal of Health Population and Nutrition [Internet]. 2015;33[1]. Available from: http://dx.doi.org/10.1186/s41043-015-0013-1

25. Mbwana HA, Kinabo J, Lambert C, Biesalski HK. Determinants of household dietary practices in rural Tanzania: Implications for nutrition interventions. Cogent Food and Agriculture [Internet]. 2016;2[1]. Available from: http://dx.doi.org/10.1080/23311932.2016.1224046

26. Iqbal S, Zakar R, Zakar MZ, Fischer F. Factors associated with infants' and young children's [6-23 months] dietary diversity in Pakistan: Evidence from the demographic and health survey 2012-13. Nutrition Journal. 2017;16[1]:1–10.

27. Sauer CM, Reardon T, Tschirley D, Liverpool-Tasie S, Awokuse T, Alphonce R, et al. Consumption of processed food & food away from home in big cities, small towns, and rural areas of Tanzania. Agricultural Economics [United Kingdom]. 2021;52[5]:749–70.

28. Ignowski L, Belton B, Tran N, Ameye H. Dietary inadequacy in Tanzania is linked to the rising cost of nutritious foods and consumption of food-away-from-home. Global Food Security [Internet]. 2023;37[February]:100679. Available from: https://doi.org/10.1016/j.gfs.2023.100679

29 Rashid, F.N., Sesabo, J.K., Lihawa, R.M. and Mkuna, E., 2024. Determinants of Household Food Expenditure in Tanzania: Implications on Food Security. Agriculture and Food Security, 13 (1), 1–16.

30. Headey, D.D., Ecker, O., Comstock, A.R. and Ruel, M.T., 2023. Poverty, price and preference barriers to improving diets in sub-Saharan Africa. *Global Food Security*, *36*, p.100664.

3

31. Elolu S, Agako A, Micheal D. Dialogues in Health Household food security , child dietary diversity and coping strategies among rural households . The case of Kole District in northern Uganda. Dialogues in Health [Internet]. 2023;3[July]:100149. Available from: https://doi.org/10.1016/j.dialog.2023.100149

32. Habibi N, Livingstone KM, Edwards S, Grieger JA. Do Older Women of Reproductive Age Have Better Diet Quality than Younger Women of Reproductive Age ? Nutrients. 2021;13[11]:3830.

33. Bhandari S, Sayami JT, Thapa P, Sayami M, Kandel BP. Dietary intake patterns and nutritional status of women of reproductive age in Nepal : findings from a health survey. Archives of Public Health [Internet]. 2016;1–11. Available from: http://dx.doi.org/10.1186/s13690-016-0114-3

34. Hidru HD, Mengesha MB, Hailesilassie Y, Welay FT. Burden and Determinant of Inadequate Dietary Diversity among Pregnant Women in Ethiopia : A Systematic Review and Meta-Analysis. Journal of Nutrition and Metabolism. 2020;2020.

35. Ahammed B, Haque R, Rahman SM, Keramat SA, Mahbub A, Ferdausi F et al. Frequency of watching television, owning a mobile phone and risk of being overweight/obese among reproductive-aged women in low- and lower-middle-income countries: A pooled analysis from Demographic and Health Survey data. Journal of Biosocial Science. 2023;55[3]:509–22.

36. Merga G, Mideksa S, Dida N, Kennedy G. Dietary diversity and associated factors among women of reproductive age in Jeldu District, West Shoa, Zone Oromia Ethiopia. Plos one. 2022;92:1–11. Available from: http://dx.doi.org/10.1371/journal.pone.0279223

37. Sekabira H, Qaim M. Can mobile phones improve gender equality and nutrition ? Panel data

evidence from farm households in Uganda. Food Policy [Internet]. 2017;73[August]:95–103. Available from: http://dx.doi.org/10.1016/j.foodpol.2017.10.004

38. Gitagia MW, Ramkat RC, Mituki DM, Termote C, Covic N, Cheserek MJ. Determinants of dietary diversity among women of reproductive age in two different agro-ecological zones of Rongai Sub-County, Nakuru, Kenya. Food and Nutrtition Research. 2019;1:1–12.

39. Nkoko N, Cronje N, Swanepoel JW, Luisa M, Gilete E. Determinants of dietary diversity for women of reproductive age [WRA] and under-five children from small-holder farming households in Lesotho. Cogent Food and Agriculture [Internet]. 2023;9[1]:1–16. Available from: https://doi.org/10.1080/23311932.2023.2231688

40. Shumayla S, Irfan EM, Kathuria N, Rathi SK, Srivastava S, Mehra S. Minimum dietary diversity and associated factors among lactating mothers in Haryana , India : a community based cross - sectional study. BMC Pediatrics [Internet]. 2022;1–9. Available from: https://doi.org/10.1186/s12887-022-03588-5

41. Tefera W, Brhanie TW, Dereje M. Dietary diversity practice and associated factors among pregnant women attending ANC in Kolfe Keranyo sub city health center , Addis Ababa, Ethiopia. 2020 May 3:2020-04.

42. Kebede AN, Sahile AT, Kelile BC. Dietary Diversity and Associated Factors Among Pregnant Women in Addis Ababa, Ethiopia, 2021. Inetrnational Journal of Public Health. 2022;67[November]:1–7.

43. Parlsca MC, Mußhoff O, Qaim M. How mobile phones can improve nutrition among pastoral communities : Panel data evidence from Northern Kenya. 2018 Jun 29.

44. Bandyopadhyay A, Haile B. Analyzing the Drivers of Household Dietary Diversity : Evidence from Burkina Faso. 2021;42[4]:530–50.

Consumption of protect and risk food groups for non-communicable diseases among women of reproductive age in rural areas of Morogoro, Tanzania

Akwilina Wendelin Mwanri[1*], Fulgence Mishili[2¶], Theresia Assenga[2¶], Suleiman Rashid[3&], Dismas Mwaseba[4&], and Susan Nchimbi-Msolla[5&]

[1]Department of Human Nutrition and Consumer Sciences, Sokoine University of Agriculture, Morogoro, Tanzania

[2]Department of Agricultural Economics and Agribusiness, Sokoine University of Agriculture, Morogoro, Tanzania

[3]Department of Food Science and Agro-processing, Sokoine University of Agriculture, Morogoro, Tanzania

[4]Department of Agric. Extension and Community Development, Sokoine University of Agriculture, Morogoro, Tanzania

[5]Department of Crop Science and Horticulture, Sokoine University of Agriculture, Morogoro, Tanzania

*Corresponding author
Email: akwmwanri@sua.ac.tz (AWM)

[¶]These authors contributed equally to this work
[&]These authors also contributed equally to this work

Abstract

Malnutrition has been persistently high in Tanzania, especially in the rural areas. Although some interventions have been implemented to reduce the rate of undernutrition, improvement is much slower than expected. Moreover, the prevalence of overweight and~~or~~ obesity among women of reproductive age has been on the increase. Changes in lifestyles specifically in eating habits may contribute to the observed increase trend ~~o~~in the prevalence of overweight and obesity. However, there are limited studies that have assessed diet quality for women. Hence, this study aimed to assess the consumption of protective and risk food groups for non-communicable diseases (NCDs) in rural areas of the Morogoro region, Tanzania. The cross-sectional study involved 512 randomly selected women of reproductive age in Mvomero district. All mothers or caretakers with children of age 6 to 23 months who resided in the area for over three months were eligible to participate. A structure questionnaire was used to collect demographic data and the Global Diet Quality Questionnaire was used to collect dietary intake data, for women~~A structured questionnaire and the Global Diet Quality Questionnaire for women were used to collect demographic and dietary intake data~~. A multivariate regression model was used to identify factors associated with consumption of diversified diets, NCD-protect, and NCD-risk food group scores. ~~The study protocol was approved by the ethics committee of the Tanzania National Health Research Institute.~~ The results show that about 42% of the women had no formal education and about one in three women own a mobile phone. About 70% consumed vegetables while 33% consumed deep fried foods. Only 34% of the women met the ~~medium~~ minimum diet diversity (MDD~~)~~-W) of five or more food groups. The mean NCD-protect score was 2.8±1.4 and the NCD-risk score was 0.77±0.97. Household income was positively associated with an increase in both protect scores and NCD-risk. Mobile phone ownership was positively associated with NCD-risk. Other factors such as age and self

Formatted: Font: (Default) Times New Roman

Formatted: Font: (Default) Times New Roman

Formatted: Not Highlight

employment in agriculture ~~are~~were positively associated NCD-protect while the number of children ~~is~~was negatively associated with NCD protect score~~, –~~. Rural communities should be educated on the importance of food diversification starting from production to consumption and the need for ~~the~~ multiple sources of income to increase purchasing power of the household while considering proper food choices to avoid the risk of NCDs. The Ministry of A~~a~~griculture and other stakeholders should support and promote production of bio~~-~~-fortified crops and keeping small animals. The ~~m~~Ministry of ~~e~~Education should ensure that school children are aware of the NCD-risk foods and make available healthier food choices around the school environment

Keywords: Diet quality, NCD-risk scores; NCD-protect scores; rural, women of reproductive age, Tanzania

Introduction

Despite the great progress made in improving food and nutrition security and reducing poverty over the past decades, undernutrition is still affecting billions of people worldwide [1]. Globally, about 170 million women are underweight; while three times as many women (610 million) are overweight [2]. Each year, about 20 million babies are born with ~~a~~ low birth weight, which is closely associated with the eating behaviors and nutrition status of women before and during pregnancy [3]. Although undernutrition affects both ~~mostly the~~ urban and rural low-income households, those in the rural areas face more challenges than their urban counterparts. This is due to social isolation, intermittent drought, limited participation of women in economic activities, limited market access, inadequate access to health care, and other socioeconomic factors such as a lower education level and a high unemployment rate [4].

Formatted: Highlight

In Tanzania, undernutrition affects infants and children under the age of five, as well as women of childbearing age. ~~in Tanzania~~ [5]. Currently, about 7.3% of non-pregnant women aged 15–49 years are underweight [6]. According to the Tanzania Demographic Health Survey and Malaria Indicator Survey, the prevalence of chronic malnutrition [stunting] among children below five years in the Morogoro region is high, at about 31% which is similar to the national average [6]. The prevalence of acute malnutrition is 4% which is above the national average and underweight ~~of~~ is 10% [6]~~.~~ Studies show that undernutrition is particularly high among low-income rural Tanzanian households, mainly because they consume more carbohydrate-rich, staple-based diets that are low in minerals and vitamins [4,7]. Other factors contributing to malnutrition include freque~~s~~nt illness, low birth weight and~~,~~ inadequate child~~-~~care practices. In addition, only 46% of rural women in Tanzania meet their minimum dietary diversity requirement (i.e., consume at least 5 food groups out of 10 daily), and women's intake of protein, fruits, and vegetables is very low [4].

Rapid urbanization, along with the global economic crisis in developing countries, has put many urban and rural people at high risk of bad quality diets and malnutrition. There is a shift from consumption of traditional diets to more refined foods high in fat, sugar and salt even in the rural areas. In fact, many studies have shown that the population of women of reproductive age is at high risk of specific micronutrient deficiencies, which can affect their health [8,9,10,11]. Generally, the diets of Tanzanian women are less diversified and have a low intake of protein, fruits and vegetables [4]. Another study by ~~[12]~~Paul et al (2022) in Tanzania found that dietary quality was inversely associated with the risk of overweight and obesity [12]. Although there is an increased prevalence of overweight or obesity among women of reproductive age from 11% in 1991 to 36% in 2022 [6], most of the reported studies concentrated on assessing nutritional status and dietary diversity with limited information on unhealthy diets. Hence this study sought to assess

Formatted: Highlight

the consumption of foods that increase the risk and those that are protective against diet-related ~~non-communicable diet-related diseases~~NCDs among rural women of reproductive age.

Materials and methods

Description of the study area

A cross-sectional study was conducted in Mvomero District, Morogoro, specifically in the rural setting. ~~, which is located at Latitude 06^{0}26' South and Longitude 37^{0}32' East.~~ Mvomero is one of the seven districts in Morogoro Region. The district is characterized by an average annual rainfall ranging between 487 and 1951 mm per year [13]. Based on the current population census, the total population for the district is 421,741, with 210,834 males and~~,~~ 210,907 females~~,~~ [14]. The district lies in three ecological zones: Miombo woodland, Highland, and mountains, as well as the Savanna River basin.

Some of the economic activities taking place in the area include agriculture, which employs about 75% of the district residents. Majority are small-scale farmers who produce various crops including maize, millet, cassava, sweet potatoes, beans, rice, sugar cane, cotton, and vegetables. Other activities done with the percentage of those involved in brackets are mining and quarrying employing (5%), trade and commerce (5%), and livestock keeping, fishing and hunting (3%). During the dry season, the majority of agricultural activities depend on the irrigation schemes established in the district which include Mkindo, Mlali, Mgeta, Ndole, Dihombo, Dakawa, and Komtonga irrigation schemes [13].

Data collection and sampling

Data were collected ~~in~~ from November to December, 2022 from six villages located in Kinda Ward, namely Kinda, Magunga, Makuyu, Matale, Ndole, and Semwali. ~~These villages are within the project's Food Hub area. The Food Hub is mainly devoted to bean farming, one of the focus crops of the FoodLAND project.~~ The sampling involved 518 randomly selected mothers with children of age between 6 and 23 months. The mothers were selected randomly from the village registry which had a list of all women with children of age 6 to 23 months. Six mothers/care takers did not complete the study because they missed the dietary intake data. All women who resided in the area for over three months were eligible to participate. Women known to be pregnant were excluded f~~or~~rom the study because there could be pregnancy induced changes in food consumption. A structured, pre-tested questionnaire translated from English to Kis~~S~~wahili was administered to collect data on socio-demographic characteristics, food availability, monthly income expenditure on food and decision-making in the household. In addition, the Global Diet Quality Questionnaire (GDQQ) – adapted for Tanzania for women ~~and for children,~~ was used to determine food group consumption, a medium dietary diversity score for women (MDD-W) and NCD-protect and NCD-risk scores based on 24-hr dietary recall [15]. The tools are available online, translated into Kiswahili and ~~it is~~are free to be used by researchers [15]. The enumerator read the examples of the foods for each food group and marked one if any of the foods on the list within the food group were consumed or zero if none were consumed.

The 29 food groups from the GDQQ were aggregated to form nine NCD-protect and eight NCD-risk ~~and nine NCD protect~~ food groups. The NCD-Protect score (0-9) was based on food consumption from nine healthy food groups, namely whole grains, pulses, nuts and seeds, vitamin A-rich orange vegetables, dark green leafy vegetables, other vegetables, vitamin A-rich fruits,

citrus, and other fruits [15]. A higher score indicates the inclusion of more health-promoting foods in the diet and correlates positively with meeting global dietary recommendations. Similarly, the The NCD-rRisk score is a score with a range from 0 to 9. The score was based on the consumption of foods from eight food groups that are recommended to limit or avoid during the past day and night (one food group, processed meat, is double-weighted). The other food groups are soft drinks (sodas), baked / grain-based sweets, other sweets, unprocessed red meat, deep-fried food, fast food, instant noodles, and packaged ultra-processed salty snacks. A higher score indicates higher consumption of foods and drinks to avoid or limit. Similarly, the NCD-Protect score (0-9) was based on food consumption from nine healthy food groups, namely whole grains, pulses, nuts and seeds, vitamin A-rich orange vegetables, dark green leafy vegetables, other vegetables, vitamin A-rich fruits, citrus, and other fruits [15]. A higher score indicates the inclusion of more health-promoting foods in the diet, and correlates positively with meeting global dietary recommendations. During analysis, six women were excluded as the reference day was a special day where they ate special foods.

Data analysis

STATA Statistical software was employed to analyze demographic factors, women's dietary diversity, and women's NCD- protect risk and risk protect scores, and the results were reported as descriptive. Some of the demographic factors used include monthly household income, a share of income spent on food, food shortage, mobile ownership, employmentself-employment in agriculture, age, education level, sex of the household head, and respondents' number of children. Categorical variables were dichotomized and coded as one for those with the characteristics and zero for those without while discrete variables such as household income and number of children werenumber of children waswere used as continuous variables.

Econometric~~s~~ model: The study used a multivariate regression model to assess the determinants of women's NCD-p~~P~~rotect and r~~R~~isk scores. The model is an extension of a simple linear regression model with the addition of more than one explanatory variable and outcome variables [16]. It can be used when the dependent variables are affected or influence by common independent variables. During analysis, ~~a~~ two~~single~~ models with two dependent variables, that is, NCD-p~~P~~rotect or ~~NCD~~ r~~R~~isk scores, ~~was~~ were analyzed with independent variables as indicated in equations 1 and 2. This study assumed that NCD-p~~P~~rotect and ~~Risk~~ risk scores can be influenced by common factors such as household monthly income, a share of income spent on food, food shortage, mobile ownership, employment, self employed in agriculture (agricultural), ~~e,~~ age, education level, sex of the household head, and respondents' number of children. The variable of household income was transformed into a log to ensure data are normally distributed.

NCD Protect~~ive~~ scores

$$= \beta_0 + \sout{Ln}B_1\text{Monthly Household Income}$$
$$+ \beta_2\text{Mother's age is aAbove35Yearsrs}$$
$$+ \beta_3\text{Mother's age is between 26 to 35 Years}$$
$$+ \beta_4\text{Share of food expenditure is Aabout 50\%}$$
$$+ \beta_5\text{Share of monthly food expenditure is Aabove 50\%}$$
$$+ \beta_6\text{NoTotal number of Children} + \beta_7\text{Secondary Education and AboveEdu1}$$
$$+ \beta_8\text{Primary Education} + \beta_9\text{ Agricultural Employement} + \beta_{10}\text{Mobile Access}$$
$$+ \beta_{11}\text{Sex of Household Head is male} + \varepsilon \ldots \ldots \ldots . . \ldots \ldots \text{(Equation1)}$$

~~NCD Risk scores~~

~~$$= \beta_0 + LnB_1Income + \beta_2Above35Yrs + \beta_3 26to35Yrs + \beta_4About50\% + \beta_5Above50\% + \beta_6No\ Children + \beta_7Edu1 + \beta_8PrimaryEdu + \beta_9AgriEmploy + \beta_{10}MobileAccess + \beta_{11}SexHHead + \varepsilon \ldots \ldots \ldots . . (Equation\ 2)$$~~

$$\begin{aligned} \text{NCD Risk} = \beta_0 &+ B_1\text{Monthly Household Income} + \beta_2\text{Mother's age is above 35Years} \\ &+ \beta_3\text{Mother's age is between 26 to 35 Years} \\ &+ \beta_4\text{Share of food expenditure is about 50\%} \\ &+ \beta_5\text{Share of ~~monthly~~ food expenditure is above 50\%} \\ &+ \beta_6\text{Total number of Children} + \beta_7\text{Secondary Education and Above} \\ &+ \beta_8\text{Primary Education} + \beta_9 s + \beta_{10}\text{Mobile Access} \\ &+ \beta_{11}\text{Sex of Household Head} + \varepsilon \ldots \ldots \ldots . . \ldots \ldots \text{(Equation 2)} \end{aligned}$$

~~Women medium~~ M~~m~~inimum dietary diversity for women

M~~inimum~~edium ~~dietary diversity score for women (MDD-W) is a proxy for micronutrient adequacy among women of reproductive age (15 to 49 years) [17,18].~~ Ten food groups that includes, "grains (white roots and tubers, and plantains), pulses (beans, peas and lentils), nuts and seeds, dairy, meat (poultry and fish), eggs, dark green leafy vegetables, other vitamin A-rich fruits and vegetables, other vegetables and fruits" were used to determine the ~~Women Medium~~ minimum ~~Dietary Diversity Score (~~MDD-W~~)~~ [17,18].. Each food was assigned a weight of 1 if consumed and 0 otherwise to give a total of 10 if all food groups were consumed in the 24 hours preceding the survey [15, 17, 18]. Women who consumed five or more food groups were regarded as attaining a minimum dietary diversity (1), while those who consumed less than five food groups

were considered otherwise (0). A binary logit regression model with a dependent variable of women's minimum dietary diversity was used to assess the influence of social--demographic factors on women's dietary diversity.

Ethical consideration

Permission to conduct the study was sought from Sokoine University of Agriculture and the regional, district, ward, and village authorities. The study was approved and ethical clearance obtained from National Health Research Ethics Committee of the Tanzania National Institute for Medical Research (NIMR) with reference number NIMR/HQ/R.8a/VOL.IX/3605. Participants were informed about the objectives of the study, the procedure, benefits, and risks. Written consent was obtained from those who agreed to participate. Participation was voluntary and each participant was free to decide otherwise at any time. All participants were identified by code numbers; data was kept in a secured computer and were accessible to the research team only.

Results

The mean age of the respondents was 26.4 ± (SD7.3), ranging from 15 to 49 years. More than 50% of the respondents were of age below 25 years. About 42% of the respondents did not have any formal education, and one in three owned a mobile phone. About 45% spent more than half of their income on food (Table 1).

Table 1: Summary of descriptive analysis for demographic and other factors (N = 512)

Variables		n(%)
Age class	15 to 25 years	284(55)
	26 to 35years	162(32)
	Above 35 years	66(13)
Share of income on food	Less than 50	142(28)
	50% Share	141(28)
	Above 50	229(45)
Mobile ownership	No	337(66)
	Yes	175(34)
Education level	No formal education	215(42)
	Primary education	265(52)
	Secondary and above	32(6)
~~Employed in A~~Agricultural	No	47(9)
	Yes	465(91)
Gender of the household head	Male household head	464(91)
	Female household head	48(9)

Descriptive results for numeric variables

The ~~average~~ mean monthly household income was 112,569TZS (47USD)~~,~~ ranging from ~~0~~ 3000TZS (1.3USD) to 1,550,000 TZS (633USD)**.** The average number of children per household was about three children, ranging from one to eleven children (Table 2).

Table 2: Descriptive results for numeric variables

Variable	Observation	Mean	Std. Dev.	Min	Max
Household ~~monthly~~ income (TZS)	512	112,569 (47USD)	138,835 (57USD)	3000TZ (1.3USD)~~0~~	1,550,000TZS (633USD)
Number of children	512	3	2	1	11

Formatted: Font: 9 pt

Formatted Table

Formatted: Font: 9 pt

Formatted: Font: 9 pt

Formatted: Font: 9 pt

Consumption of NCD-protect~~ive~~ and NCD-risk food groups (N = 512)

Table 3 shows consumption of food groups that are considered healthy or unhealthy for women. Regarding healthy foods, vegetables and pulses were commonly consumed by a large proportion of women (72% and 44%), respectively. However, only 16% of women consumed whole grains. Among the unhealthy foods, relatively high proportion of women consumed deep fried foods (33%), and baked/grain based sweet ~~baked~~ foods (15%) were consumed as unhealthy food.

Table 3: Consumption of NCD-protect~~ive~~ and NCD-risk food groups (N = 512)

NCD Protect food groups	n(%)	NCD Risk food groups	n(%)
Whole grains	82(16)	Sugar sweetened beverages	28(6)
Pulse	226(44)	Baked/~~G~~grains based sweet~~s baked foods~~	77(15)
Nuts and seeds	54(11)	Other sweet	45(9)
Vitamin A rich orange vegetables	16(3)	Processed meat	0(0)
Dark green leafy vegetables	361(71)	Unprocessed red meat	38(7)
Other vegetables	370(72)	Deep-fried foods	168(33)
Vitamin A rich fruits	126(25)	Fast foods and instant noodles	2(0.03)
Citrus	17(3)	Processed packed salty snacks	2(0.03)
Other fruits	164(32)		

Descriptive analysis for women NCD Protect~~ive~~ and Risk scores

The mean NCD-protect~~ive~~ score was 2.8±1.4, with a range from 0 to 8 food groups ~~. Regarding the~~while the NCD-risk score ~~for women, it was~~ around 0.7~~7~~ ±0.9~~7~~ with a range of 0 to 5 food groups (Table 4~~a~~). ~~The mean NCD-risk score was higher among women with a single child (0.9±1.0)~~

~~4(a): Women NCD Protect and Risk Score summary~~

~~Women NCD Protect and Risk score~~	~~N~~	~~Mean~~	~~S.D~~	~~Min~~	~~Max~~
~~NCD Protect score~~	~~512~~	~~2.8~~	~~1.4~~	~~0~~	~~8~~
~~NCD Risk score~~	~~512~~	~~0.7~~	~~0.9~~	~~0~~	~~5~~

Formatted Table

~~-~~The mean NCD protect~~ive~~ score was higher among women who spent about half of their income on food (mean 3.0 ±1.5) and~~.~~ higher among women with a single child (0.9±1.0) ~~Regarding the NCD-risk score for women, it was around 0.77 ±0.97 with a range of 0 to 5 food groups. The mean NCD-risk score was higher among women with a single child (0.9±1.0)~~ (Table 4~~b~~).

Table 4 ~~(b)~~: Descriptive analysis for women NCD ~~Protect~~ protect (mean+/-SD) and ~~Risk~~NCD--risk (mean+/-SD) Scores

Variables		Observations	*NCD-Protect	*NCD-Risk
Age class	15 to 25 years	282	2.8±1.4	0.8±0.96
	26 to 35 years	163	2.7±1.5	0.7±1.1
	Above 35 years	67	2.6±1.4	0.5±0.8
Number of children	1 child	161	3.0±1.5	0.9±1.0
	2 to 3 children	208	2.7±1.4	0.7±1.0
	Above 3 children	143	2.6±1.4	0.6±1.0

Formatted Table

		Less than 50%	142	2.9±1.6	0.8±1.0
	Share of income on food	About 50%	141	3.0±1.5	0.76±0.9
		Above 50%	229	2.6±1.3	0.6±0.98
		No	337	2.7±1.5	0.6±0.9
	Mobile ownership	Yes	175	2.9±1.4	0.9±1.1
		No formal education	215	2.7±1.4	0.7±1.0
	Education level	Primary education	266	2.8±1.5	0.7±1.0
		Secondary and above	31	2.7±1.3	0.9±0.9
~~Employed in~~ agricultural	No	47	2.4±1.3	0.7±0.8	
		Yes	465	2.8±1.5	0.7±1.0
	Gender of the household head	Male household head	463	2.8±1.4	0.7±1.0
		Female household head	49	2.4±1.4	0.7±0.8
	Total			2.8± 1.4	0.71±0.97

*Value in the table mean ± standard deviation

Influence of demographic factors to women NCD- protect and NCD-risk scores.

The multivariate regression model shows that there was a significant association between the NCD-protect score and household income, age (above 35 years), number of children in the household, agricultural employment, and sex of the household head. There was a significant positive association between household income and mobile phone ownership with NCD-risk score (Table 5).

Table 5: Influence of demographic factors to women NCD- Protect and risk scores

	NCD Protect~~ive~~			NCD Risk		
**Variables**	**Coefficients**	**S.E**	**P>t**	**Coefficients**	**S.E**	**P>t**

Formatted Table

Formatted: Highlight

Household income (Log)	0.299	0.059	0.000***	0.254	0.040	0.000***
Above 35 years	0.784	0.316	0.013**	-0.043	0.213	0.841
Age 26 to35 years	0.293	0.184	0.112	0.012	0.124	0.923
About 50% share of income on food	0.168	0.171	0.326	0.052	0.115	0.65
Above 50% share of income on food	-0.248	0.152	0.103	-0.142	0.102	0.165
Number of children	-0.205	0.057	0.000***	-0.041	0.039	0.285
Secondary education and above	-0.198	0.297	0.506	0.043	0.200	0.83
Primary Education	0.063	0.136	0.646	-0.142	0.092	0.123
Agricultural Employment	0.458	0.226	0.043**	0.108	0.152	0.48
Mobile Ownership	0.084	0.141	0.55	0.211	0.095	0.027**
Sex of Household head	0.369	0.222	0.097*	0.016	0.150	0.916
Constant	-0.901	0.724	0.214	-2.044	0.488	0.000

~~*** Significant at 1%, ** Significant at 5% and * Significant at 10%.~~

Equation	RMSE	R-sq	F
NCD Protective	1.39740	0.101	4.90**893
NCD Risk	0.942	0.1109	5.35**47

*** Significant at 1%, ** Significant at 5% and * Significant at 10%.

Formatted Table

Formatted: Left

Formatted: Left

Minimum dietary diversity for women and its determinants

Tables 6 (a) and (b) 7 show that only one-third of the women (34%, n=174) attained the minimum dietary diversityMDD-W. Household income, age of the mother, number of children, ownership of a mobile phone and sex of the household head were associated with the MDD-W

Table 6 (a): Women mMinimum dietary diversity scorefor women

~~Women dietary diversity~~	~~N~~	~~%~~

DDS<5	338	66
DDS≥5	174	34

Women dietary diversity	N	%	Mean	S.D	Min	Max
DDSMDD-W<5	338	66	3	1	1	4
DDSMDD-W≥5	174	34	6	1	5	10

Formatted Table

Table 6 (b)7: Determinants of women minimum dietary diversity

Variables	Coefficients	Robust S.E	P>z
Household income (log)	0.522	0.096	0.000***
Above 35 years	1.346	0.532	0.011**
Age 26 to 35 years	0.560	0.300	0.062*
About 50% share of income on food share	0.085	0.272	0.755
Above 50% share of income on food	-0.396	0.244	0.105
Number of children	-0.345	0.100	0.001***
Secondary education and Above	-0.003	0.457	0.994
Primary Education	0.182	0.221	0.41
Agricultural Employment	0.608	0.370	0.1
Mobile Ownership	0.570	0.225	0.011**
Sex of household head	0.962	0.387	0.013**

~~Constant~~	~~-7.484~~	~~1.184~~	~~0~~

Independent Variables	Odds Ratio	Robust S.E	P>z	95% Conf.Interval	
Household income (log)	1.6847	0.1615	0.000***	1.3961	2.0330
Above 35 years	3.8429	2.0441	0.011**	1.3549	10.8999
Age 26 to 35 years	1.7504	0.5243	0.062	0.9732	3.1485
About 50% share of income on food share	1.0886	0.2956	0.755	0.6393	1.8535
Above 50% share of income on food	0.6727	0.1643	0.105	0.4168	1.0858
Number of children	0.7084	0.0710	0.001***	0.5820	0.8623
Secondary education and Above	0.9968	0.4552	0.994	0.4073	2.4395
Primary Education	1.1997	0.2652	0.41	0.7779	1.8503
Agricultural ~~Employment~~	1.8369	0.6798	0.1	0.8894	3.7939
Mobile Ownership	1.7683	0.3981	0.011**	1.1374	2.7490
Sex of household head	2.6168	1.0126	0.013**	1.2257	5.5866
Constant	0.0006	0.0007	0.000	0.0001	0.0057

*** Significant at 1%, ** Significant at 5% and * Significant at 10%.

Discussion

This study aimed to assess the consumption of food groups that increase the risk and those that are protective against ~~non-communicable diet-related diseases~~diet related NCDs among women of reproductive age in rural areas of Morogoro, Tanzania. ~~To the best of our knowledge, this is the first study that use the Global Diet Quality Questionnaire (GDQQ) to estimate medium dietary diversity, NCD-protect and NCD-risk scores among women of reproductive age in Tanzania [based on 24-hr dietary recall] [15].~~There are limited number of studies that used GDQQ to estimate minimum dietary diversity, NCD-protect and NCD-risk scores among women of reproductive age in Tanzania [based on 24-hr dietary recall] [15]. The findings of this study indicate that the mean NCD-protect and NCD-risk scores for women were low, but the mean NCD-protect score was higher than that of the ~~NCD-~~risk score, implying that rural women are more

Formatted: Font: (Default) Times New Roman, 11 pt, Bold
Formatted Table
Formatted: Font: (Default) Times New Roman, 11 pt
Formatted: Font: (Default) Times New Roman, 11 pt
Formatted: Font: (Default) Times New Roman, 11 pt
Formatted: Font: (Default) Times New Roman, 11 pt
Formatted: Font: (Default) Times New Roman, 11 pt
Formatted: Font: (Default) Times New Roman, 11 pt
Formatted: Font: (Default) Times New Roman, 11 pt
Formatted: Font: (Default) Times New Roman, 11 pt
Formatted: Font: (Default) Times New Roman, 11 pt
Formatted: Font: (Default) Times New Roman, 11 pt
Formatted: Font: (Default) Times New Roman, 11 pt
Formatted: Font: (Default) Times New Roman, 11 pt

protected from NCDs as far as food intake is concerned. The lower consumption of NCD-risk food groups observed in this study can possibly be due to the fact that women living in rural areas have limited access and low purchasing power and hence depend mainly on what they produce. This finding is consistent with a review done by Akumu et al [19], which shows that, the diet in Uganda is mainly composed of starch staples, especially roots, tubers, bananas, and legumes which are healthy foods. A study conducted in Peru among women of reproductive age reported a higher prevalence of consumption of unhealthy foods, such as sugar-sweetened beverages, consumption in by 97% [20].

A high proportion of women consumed vegetables and pulses in the healthy food groups. The observed practice is not surprising because the villages involved in the study produce pulses and plenty of vegetables in the river valleys. This finding is in line with the one reported in a study conducted in rural areas of Kenya, which revealed that vegetables and pulses were mostly consumed by women in rural areas [21]. However, contrasting results have been shown in other studies with a shortfall of fruits and vegetable intake in the diet of women of reproductive age [22,23,24]. Nonetheless, the findings of this study are inconsistent with several other studies that reported that, the most commonly consumed food groups by women of reproductive age in rural areas were staple foods such as [grains or cereal], roots and tubers [25,23,26]. These results can possibly be explained by seasonality, socioeconomic variation among the study groups, as well as the availability of these food groups in households where vegetables are commonly produced and accessed from the market at a low price. However, the reported consumption was qualitative hence not necessarily imply that the studied women meet recommended amount of vegetable intake.

Deep-fried foods and baked/grain-baseds sweets baked foods were the most commonly consumed unhealthy foods. The high consumption of deep-fried foods shows changes in food preparation

methods from common boiling or steaming to deep frying due to the availability and access of cooking oil, ~~as well as~~and shifts in dietary preferences, taste/palatability and lifestyles. Other studies conducted in Tanzania reported similar findings that about 21% of the rural households consume ultra-processed foods and foods away from home which are high in fat, sugar and ~~,~~ salt ~~and fat~~ [27,28].

From regression analysis, household income was positively associated with both NCD-protect and NCD-risk scores. This means households with more income can access more diversified diets, with some being in the healthy and others in the unhealthy groups. Higher-income households often have more disposable income, making it easier for them to afford a wide variety of foods, including healthier and unhealthy options. Nevertheless, lower-income households often face greater challenges in accessing and affording healthier food options, which can lead to consumption of monotonous, less diversified foods based on what they produce or what they can afford. More food access and availability due to high income may not necessarily lead to healthier option. ~~[27]~~ It was reported that nutritious foods eaten at home in Tanzania has not improved with rising of income [27]. ~~.~~ A study done in ~~the~~Tanzania using Household Budget Survey ~~USA support~~data support the current findings that lower income households purchase fewer healthful foods compared with higher income households [29]. Another study conducted in the ~~United Kingdom~~in Sub-Saharan Africa reported similar findings; lower food expenditure is likely to be a key contributor to less healthy food choices among lower socioeconomic groups [30].

In this study, being older than 35 years was associated with greater consumption of NCD-protect food groups than their younger counterparts. With age, people often make lifestyle changes to prioritize health and well-being, including dietary modifications, as they develop more established and health-conscious dietary habits, which can include a greater focus on consuming NCD-

protective foods [31]. It could also be explained by their being more exposed to nutrition education compared to the younger women. Another reason might be health conditions, as older individuals may have pre-existing health conditions that necessitate dietary changes, encouraging the consumption of NCD-protective foods. The finding of the current study is in line with a study conducted in Australia and Nepal, which found that being older than 35 years had higher component scores in limiting consumption of NCD-risk food groups, such as limiting consumption of saturated fat and consuming low-fat milk [32,33].

Furthermore, there were a significant negative association between the NCD-protect score and the number of children in the households. This means households with fewer children had higher NCD-protect scores compared to those with more children. This implies that households with few children are more likely to diversify their diet. These findings are supported by results from a systematic review and meta-analysis conducted in Ethiopia, which reported that, pregnant women living in larger families were more likely to consume less diversified diets compared to those living in smaller families [34]. This can possibly be explained by the fact that families with more children may have to allocate their resources, including food, among a greater number of individuals, which could affect the quality and diversity of their diet and strain the economic resources of a household, limiting their ability to purchase a variety of NCD-protect foods. It might as well be related to time constraints that make it more challenging to prepare and consume healthier meals, potentially leading to reliance on more convenient but less nutritious options. Despite this, smaller families might have more time to plan and prepare nutritious meals, leading to increased consumption of healthy foods.

Having a mobile phone was significantly associated with NCDs-risk scores. Mobile phones enable exposure to digital marketing and advertising, potentially influencing food choices and

consumption patterns [35]. Additionally, women who own mobile phones may be more influenced by friends or family members to consume unhealthy foods. They could also be doing business outside the home, hence purchasing unhealthy foods sold around by the food vendors, which are mainly f~~F~~rench fries, African doughnuts, and fried bananas or cassava. Findings of this study are inconsistent with those reported in a study conducted in Ethiopia and Uganda, which revealed that owning a mobile phone was significantly associated with a wide intake of a variety of food groups due to access to nutrition and health information [36,37].

Factors associated with ~~medium~~ minimum dietary diversity for women of reproductive age

Regression analysis indicated that being older was associated with MDD-W. This can possibly be explained by the fact that older individuals often have more diverse food preferences and culinary experiences accumulated over time [38]. Economic stability and access to a wider range of foods may also increase with age, allowing older women to have more opportunities to diversify their diets. Findings of this study are in line with the studies conducted in Lesotho, Kenya and India ~~[39,40] respectively,~~ which reported older individuals having a higher minimum dietary diversity compared to their younger counterparts [39,40]. Additionally, as people age, they may develop a greater awareness of the importance of a varied diet for overall health and well-being. It is also possible that older people may have more income from the economic activities they have established over the years than the younger ones which enable then to diversify foods more. They may also receive some support from their children who are working outside the village.

Higher-income households can contribute to greater dietary diversity due to increased access to a

variety of nutritious foods. High-income households often have the financial means to purchase a wider range of fruits, vegetables, proteins, and other essential nutrients, promoting a more diverse and balanced diet [41]. Additionally, economic stability reduces food insecurity, allowing for consistent access to different food groups, which is crucial for maintaining nutrition status. The findings of this study are supported by a study in Addis Ababa, Ethiopia [42] and Lesotho [39].

Having a mobile phone in rural areas among women could be associated with higher dietary diversity due to improved access to information about nutrition, agricultural practices, and market prices. Mobile phones enable women to receive timely updates on farming techniques, access weather forecasts, and connect with markets, allowing them to make informed decisions about crop choices and food purchases [43]. The findings of this study are in line with the study conducted in Jerdu, Ethiopia by [36] which found that women who own mobile phones were more likely to have a minimum dietary diversity score than their counterparts. Moreover, mobile phones can facilitate communication within communities, creating opportunities for knowledge-sharing related to diverse and nutritious food options among women.

Minimum dietary diversity score for women was higher in male headed households than female headed ones. This can possibly be explained by male heads of households may have greater access to resources, which can facilitate the purchase of healthier food options and wide variety of foods [1]. It might as well be related to the demand of household chores which may limit the time and energy to plan and prepare a wide variety of foods in female headed households. Findings of this study are supported by the study done in Tanzania and Burkina Faso, which found that ~~minimum dietary diversity~~ MDD-W was lower in female headed households than their counterparts [1,44].

In addition, number of children in the households was inversely associated with minimum dietary diversity. This finding can possibly be explained by the fact that a smaller number of children in

households may lead to greater dietary diversity, there being more resources available per child, allowing for a wider range of food options. Additionally, smaller families might be more flexible in adapting to diverse food preferences and accommodating a wider range of foods. This contrasts with larger families, where resource constraints and time limitations might result in a more limited variety of foods. Studies done in Nakuru county Kenya and Lesotho reported a similar finding that household size can also influence adequate dietary diversity among women of reproductive age [38,39].

Conclusion

Generally, the mean NCD-protect score was higher than the NCD-risk score, implying that rural women are more protected from NCDs as far as food intake is concerned. Although the mean NCD-risk was low, about one out of three women consumed deep-fried foods in the day preceding the survey. Household income, mobile ownership, age, sex of the household head, and respondents' number of children were strongly associated with NCD-~~ P~~protect and r~~R~~isk scores. Only one in three women met the recommended MDD-W implying that majority may have inadequate micronutrient intake. Therefore, rural communities should be supported to diversify food production and sources of income to increase purchasing power while considering proper food choices to increase micronutrients intake and avoid the risk for NCDs. The Ministry of A~~a~~griculture and other stakeholders should support and promote production of bio fortified crops and keeping small animals. The M~~m~~inistry of E~~e~~ducation should ensure that school children are aware of the NCD-risk foods and make available healthier foods choices around the school environment

It is important to note that the study was conducted in one region (Morogoro) out of 31 regions and on one district (Mvomero) out of the eight districts of Morogoro region, Tanzania; hence the data may not be generalized to other regions due to ~~variations across~~variations across the regions.

Acknowledgements

We acknowledge the study participants for accepting to participate in the study. We appreciate the support from the field assistants and we thank the regional, district and Sokoine University of Agriculture authorities for providing support to carry out the study**.** We acknowledge support from FoodLAND project members who contributed to this study in one way or the other.

~~Source of funds~~

~~This research is part of the FoodLAND project that has received funding from the European Union's Horizon 2020 research and innovation programme under grant agreement [GA No 862802]. The views and opinions expressed in this document are the sole responsibility of the authors and do not necessarily reflect the views of the European Commission. The funders had no role in study design, data collection and analysis, decision to publish, or preparation of the manuscript~~.

References

1. Ochieng J, Afari-Sefa V, Lukumay PJ, Dubois T. Determinants of dietary diversity and the potential role of men in improving household nutrition in Tanzania. PLoS One. 2017;12[12]:1–18.
2. United Nations Children's Fund. UNICEF Programming Guidance. Prevention of malnutrition in women before and during pregnancy and while breastfeeding [Internet]. Caring for Children Born Small for Gestational Age. New York; 2021. Available from: https://www.unicef.org/media/115361/file/Maternal
3. United Nations Children's Fund and World Health Organization. Low Birthweight Estimates: Levels and trends 2000-2015. Vol. 7, The Lancet Global Health. Geneva; 2019.
4. Madzorera I, Blakstad MM, Bellows AL, Canavan CR, Mosha D, Bromage S, et al. Food Crop Diversity, Women's Income-Earning Activities, and Distance to Markets in Relation to Maternal Dietary Quality in Tanzania. Journal of Nutrition [Internet]. 2021;151[1]:186–96. Available from: https://doi.org/10.1093/jn/nxaa329
5. Ministry of Health, Community Development, Gender, Elderly and Children [MoHCDGEC] [Tanzania Mainland], Ministry of Health [MoH] [Zanzibar], Tanzania Food and Nutrition Centre [TFNC], National Bureau of Statistics [NBS], Office of the Chief Government Statistician and U. Tanzania National Nutrition Survey using SMART Methodology [TNNS] 2018. Dar es Salaam, Tanzania;2019.
6. Ministry of Health [MoH] [Tanzania Mainland], Ministry of Health [MoH] [Zanzibar], National Bureau of Statistics [NBS], Office of the Chief Government Statistician [OCGS], and ICF. Tanzania Demographic and Health Survey and Malaria Indicator Survey 2022 Key Indicators Report. 2023. Availablefrom:https://www.nbs.go.tz/nbs/takwimu/dhs/Tanzania_DHS-MIS_2022_Final_Report.pdf
7. Huang M, Sudfeld C, Ismail A, Vuai S, Ntwenya J, Mwanyika-Sando M, et al. Maternal Dietary Diversity and Growth of Children Under 24 Months of Age in Rural Dodoma, Tanzania. Food

Nutrition Bulletin. 2018;39[2]:219–30.

8. Brazier AKM, Lowe NM, Zaman M, Shahzad B, Ohly H, McArdle HJ, et al. Micronutrient status and dietary diversity of women of reproductive age in rural Pakistan. Nutrients. 2020;12[11]:1–15.

9. Chaudhary SR, Govil S, Lala MK, Yagnik HB. Infant and young child feeding index and its association with nutritional status: A cross-sectional study of urban slums of Ahmedabad. Journal of Family and Community Medicine. 2018;25[2]:88–94.

10. Agada M, Igbokwe E. Dietary Diversity of Rural Households in North Central Nigeria. European Journal of Nutrition and Food Safety. 2015;5[3]:150–5.

11. Nupo SS, Oguntona CRB, Onabanjo OO, Fakoya EO. Dietary diversity scores and nutritional status of women in two seasons in rural areas of Ogun State, Nigeria. Nutrition Food Science. 2013;43[1]:60–7.

12. Paulo HA, Mosha D, Mwanyika-Sando M, Mboya IB, Madzorera I, Killewo J, et al. Role of dietary quality and diversity on overweight and obesity among women of reproductive age in Tanzania. PLoS One [Internet]. 2022;17[4 April]:1–13. Available from: http://dx.doi.org/10.1371/journal.pone.0266344

13. United Republic of Tanzania. Mvomero district investment profile. 2017. Available from: https://mvomerodc.go.tz/storage/app/uploads/public/5bb/da6/b9b/5bbda6b9b955b438674778.pdf

14. United Republic of Tanzania. Administrative Units Population Distribution Report. National Population and House Census of Tanzania. National Bureau of Statistics, Dar es Salaam, Tanzania.2022.Availablefrom:https://www.nbs.go.tz/nbs/takwimu/Census2022/Administrative_units_Population_Distribution_Report_Tanzania_volume1a.pdf

15. Global Diet Quality Project. DQQ for Tanzania. Accessed at dietquality.org https://www.dietquality.org/tools and https://www.dietquality.org/countries/tza

16. Hidalgo B, Goodman M. Multivariate or multivariable regression? American Journal of Public Health. 2013;103[1]:39–40.

17. Martin-Prevel Y, Arimond M, Allemand P, Wiesmann D, Ballard TJ, Deitchler M, et al.

Development of a dichotomous indicator for population-level assessment of dietary diversity in women of reproductive age. Current Development Nutrition. 2018;1[12].

18. Food and Agriculture Organization, 360 FHI. Minimum Dietary Diversity for Women: A Guide for Measurement [Internet]. 2016. 176 p. Available from: http://www.fao.org/3/a-i5486e.pdf

19. Akumu G, Ogenrwoth B, Mugisha J, Muyonga JH. Dietary Patterns in Uganda and Their Influencing Factors: a Critical Review. African Journal of Food, Agriculture, Nutrition and Development. 2023;23[2]:22328–53.

20. Pradeilles R, Pareja R, Creed-Kanashiro HM, Griffiths PL, Holdsworth M, Verdezoto N, et al. Diet and food insecurity among mothers, infants, and young children in Peru before and during COVID-19: A panel survey. Maternal and Child Nutrition. 2022;18[3].

21. Kishino M, Hirose M, Hida A, Tada Y, Ishikawa-Takata K, Hara K, et al. Characteristics of Dietary Intake in Relation to the Consumption of Home-Produced Foods among Farm Women in Two Rural Areas of Kenya: A Preliminary Study. Dietetics. 2022;1[3]:242–54.

22. Otunchieva A, Smanalieva J, Ploeger A. Dietary Quality of Women of Reproductive Age in Low-Income Settings: A Cross-Sectional Study in Kyrgyzstan. Nutrients. 2022;14[2].

23. Keno S, Bikila H, Shibiru T, Etafa W. Dietary diversity and associated factors among children aged 6 to 23 months in Chelia District, Ethiopia. BMC Pediatrics. 2021;21[1]:1–8.

24. Naicker A, Venter CS, MacIntyre UE, Ellis S. Dietary quality and patterns and non-communicable disease risk of an Indian community in KwaZulu-Natal, South Africa. Journal of Health Population and Nutrition [Internet]. 2015;33[1]. Available from: http://dx.doi.org/10.1186/s41043-015-0013-1

25. Mbwana HA, Kinabo J, Lambert C, Biesalski HK. Determinants of household dietary practices in rural Tanzania: Implications for nutrition interventions. Cogent Food and Agriculture [Internet]. 2016;2[1]. Available from: http://dx.doi.org/10.1080/23311932.2016.1224046

26. Iqbal S, Zakar R, Zakar MZ, Fischer F. Factors associated with infants' and young children's [6-23 months] dietary diversity in Pakistan: Evidence from the demographic and health survey 2012-13. Nutrition Journal. 2017;16[1]:1–10.

27. Sauer CM, Reardon T, Tschirley D, Liverpool-Tasie S, Awokuse T, Alphonce R, et al. Consumption of processed food & food away from home in big cities, small towns, and rural areas of Tanzania. Agricultural Economics [United Kingdom]. 2021;52[5]:749–70.

28. Ignowski L, Belton B, Tran N, Ameye H. Dietary inadequacy in Tanzania is linked to the rising cost of nutritious foods and consumption of food-away-from-home. Global Food Security [Internet]. 2023;37[February]:100679. Available from: https://doi.org/10.1016/j.gfs.2023.100679

~~29. French SA, Tangney CC, Crane MM, Wang Y, Appelhans BM. Nutrition quality of food purchases varies by household income: The Shopper study. BMC Public Health. 2019;19[1]:1–7.~~

29 Rashid, F.N., Sesabo, J.K., Lihawa, R.M. and Mkuna, E., 2024. Determinants of Household Food Expenditure in Tanzania: Implications on Food Security. Agriculture and Food Security, 13 (1), 1–16.

30. Headey, D.D., Ecker, O., Comstock, A.R. and Ruel, M.T., 2023. Poverty, price and preference barriers to improving diets in sub-Saharan Africa. *Global Food Security*, *36*, p.100664.

3~~0. Pechey R, Monsivais P. Socioeconomic inequalities in the healthiness of food choices : Exploring the contributions of food expenditures. Preventive Medicine [Baltim] [Internet]. 2016;88:203–9. Available from: http://dx.doi.org/10.1016/j.ypmed.2016.04.012~~

31. Elolu S, Agako A, Micheal D. Dialogues in Health Household food security , child dietary diversity and coping strategies among rural households . The case of Kole District in northern Uganda. Dialogues in Health [Internet]. 2023;3[July]:100149. Available from: https://doi.org/10.1016/j.dialog.2023.100149

32. Habibi N, Livingstone KM, Edwards S, Grieger JA. Do Older Women of Reproductive Age Have Better Diet Quality than Younger Women of Reproductive Age ? Nutrients. 2021;13[11]:3830.

33. Bhandari S, Sayami JT, Thapa P, Sayami M, Kandel BP. Dietary intake patterns and nutritional status of women of reproductive age in Nepal : findings from a health survey. Archives of Public Health [Internet]. 2016;1–11. Available from: http://dx.doi.org/10.1186/s13690-016-0114-3

34. Hidru HD, Mengesha MB, Hailesilassie Y, Welay FT. Burden and Determinant of Inadequate

Formatted: Font: (Default) Arial, 10 pt, Font color: Custom Color(RGB(34,34,34)), Check spelling and grammar, Pattern: Clear (White)

Dietary Diversity among Pregnant Women in Ethiopia : A Systematic Review and Meta-Analysis. Journal of Nutrition and Metabolism. 2020;2020.

35. Ahammed B, Haque R, Rahman SM, Keramat SA, Mahbub A, Ferdausi F et al. Frequency of watching television, owning a mobile phone and risk of being overweight/obese among reproductive-aged women in low- and lower-middle-income countries: A pooled analysis from Demographic and Health Survey data. Journal of Biosocial Science. 2023;55[3]:509–22.

36. Merga G, Mideksa S, Dida N, Kennedy G. Dietary diversity and associated factors among women of reproductive age in Jeldu District, West Shoa, Zone Oromia Ethiopia. Plos one. 2022;92:1–11. Available from: http://dx.doi.org/10.1371/journal.pone.0279223

37. Sekabira H, Qaim M. Can mobile phones improve gender equality and nutrition ? Panel data evidence from farm households in Uganda. Food Policy [Internet]. 2017;73[August]:95–103. Available from: http://dx.doi.org/10.1016/j.foodpol.2017.10.004

38. Gitagia MW, Ramkat RC, Mituki DM, Termote C, Covic N, Cheserek MJ. Determinants of dietary diversity among women of reproductive age in two different agro-ecological zones of Rongai Sub-County, Nakuru, Kenya. Food and Nutrtition Research. 2019;1:1–12.

39. Nkoko N, Cronje N, Swanepoel JW, Luisa M, Gilete E. Determinants of dietary diversity for women of reproductive age [WRA] and under-five children from small-holder farming households in Lesotho. Cogent Food and Agriculture [Internet]. 2023;9[1]:1–16. Available from: https://doi.org/10.1080/23311932.2023.2231688

40. Shumayla S, Irfan EM, Kathuria N, Rathi SK, Srivastava S, Mehra S. Minimum dietary diversity and associated factors among lactating mothers in Haryana , India : a community based cross - sectional study. BMC Pediatrics [Internet]. 2022;1–9. Available from: https://doi.org/10.1186/s12887-022-03588-5

41. Tefera W, Brhanie TW, Dereje M. Dietary diversity practice and associated factors among pregnant women attending ANC in Kolfe Keranyo sub city health center , Addis Ababa, Ethiopia. 2020 May 3:2020-04.

42. Kebede AN, Sahile AT, Kelile BC. Dietary Diversity and Associated Factors Among Pregnant Women in Addis Ababa, Ethiopia, 2021. Inetrnational Journal of Public Health. 2022;67[November]:1–7.

43. Parlsca MC, Mußhoff O, Qaim M. How mobile phones can improve nutrition among pastoral communities : Panel data evidence from Northern Kenya. 2018 Jun 29.

44. Bandyopadhyay A, Haile B. Analyzing the Drivers of Household Dietary Diversity : Evidence from Burkina Faso. 2021;42[4]:530–50.

Response to reviewers' comments: Dec 2024

Dear Editor,

We appreciate the comments and suggestions given by the reviewers. We also honour the comments of the editor that were very pertinent to improve the language, clarity and understanding to the readers.

We hereby report the revisions made and submit for your considerations

**Reviewers comments**	**Response**
Please state what role the funders took in the study. If the funders had no role, please state: "The funders had no role in study design, data collection and analysis, decision to publish, or preparation of the manuscript."<br>If this statement is not correct you must amend it as needed.<br>Please include this amended Role of Funder statement in your cover letter; we will change the online submission form on your behalf.	The role of the funders is stated in the submission letter. It is also included in source of funds section in the manuscript
When completing the data availability statement of the submission form, you indicated that you will make your data available on acceptance. We strongly recommend all authors decide on a data sharing plan before acceptance, as the process can be lengthy and hold up publication timelines. Please note that, though access restrictions are acceptable now, your entire data will need to be made freely accessible if your manuscript is accepted for publication. This policy applies to all data except where public deposition would breach compliance with the protocol approved by your research ethics board. If you are unable to adhere to our open data policy, please kindly revise your statement to explain your reasoning and we will seek the editor's input on an exemption. Please be assured that, once you have provided your new statement, the assessment of your exemption will not hold up the peer review process.	The data will be made available. The project consortium is aware of the requirement and ready to share data when needed
Please amend either the abstract on the online submission form (via Edit Submission) or the abstract in the manuscript so that they are identical.	Amendment of the abstract was done to make the one online identical with the manuscript
Your ethics statement should only appear in the Methods section of your manuscript. If your ethics statement is written in any section besides the Methods, please move it to the Methods section and delete it from any other section. Please ensure that your ethics statement is included in your manuscript, as the ethics statement entered into the online submission form will not be published alongside your manuscript.	The ethics statement in the abstract was deleted

The results are largely consistent with the nationally representative survey conducted in 2021 by Gallup Poll of 1000 women and men in both urban and rural areas of Tanzania, although the authors of this study do not seem to be aware of that data.	We appreciate the comment on the additional manuscript published in 2021. We included it to improve our discussion
What time of year the data were collected (especially with respect to seasonal food availability),<br><br>how random sampling of the women across the villages was carried out, and<br><br>who conducted the data collection (number of people, training provided, any assessment of inter-rater reliability).<br><br>With respect to the results, it would be helpful to have a full description of the MDD-W results by dependent variable (Table 6a) - see my suggestions in the attached PDF file. Furthermore, the results of the logistic regression model used to explore the association of the dependent variables with MDD-W would be easier to interpret if the coefficients were presented as odds ratios with 95% confidence intervals	The mothers were selected randomly from the village registry which had a list of all women with children of age 6 to 23 months<br><br>10 enumerators (all females) were trained to collect data. The data collection tool was pretested and checking for consistency in asking questions.<br>Table 6 on the MDD-W was improved to include OR and CI as suggested (Table 6b); Pg 16
I noted that the minimum dietary diversity indicator is frequently incorrectly described as 'medium dietary diversity' throughout the manuscript. Also, there were several instances where the in-text citation of a study was done incorrectly. Please add the name of the study author when referring to an article in the text rather than saying "a study done by [19]" - example from line 247.	Thank you for the observation. We admit this incorrect way of citation. It was corrected throughout the manuscript
In both the results and discussion sections, I found that it would be helpful to consider these women more holistically, looking at patterns or groups within the dataset. For example, rather than looking at independent associations of age and mobile phone ownership, were there patterns across these characteristics? Were the women who owned mobile phones also more likely to be younger women? Similarly for the discussion of NCD-protect and MDD-W, there is a large amount of overlap in these two metrics of diet quality. While they are distinct indicators designed to be interpreted in	It is true that the women who owned mobile phones were younger. Generally, 50% of those of age 15 to 25 had mobile phones compared to 36% among 26 to 35 years and 14% for those older than 35 years<br><br>However, all this variables were entered in the model together to

different ways, there did not seem to be a recognition that the same dietary intake patterns that drive one also drive the other. Rather than discussing them separately, it would be helpful to consider what are the common underlying factors that make it difficult for women to consume more fruits and vegetables, for example.<br><br>Finally, I found the discussion of implications of these results a bit sparse, limited to one sentence in the Conclusion section. Rather than placing all the responsibility on these women and their communities for increased production, increased incomes and better food choices, what are the potential implications for agriculture, food and healthy diet policies in the country? Given that households may start to purchase more food as incomes increase, what are the options for ensuring that more diverse and healthy foods are available for purchase in this district, even if it is not possible for the communities to produce all of it themselves? What role can public health actors play in providing nutrition education to all ages, including young people, to enhance awareness of foods that are associated with increased risk of NCDs? And given the challenge in meeting nutrient adequacy with locally available foods, how can the country's food fortification agenda be scaled up to benefit women in these communities? These are some possible questions for the authors to consider as they expand a bit more on what can be done in response to these findings.	control for confounding effect. The discussion section was revised as suggested<br><br>We appreciate the comment. The conclusion was revised as suggested
The conclusion here appears to put the onus on rural communities to increase their knowledge (education) about diet diversification, increased income and healthy food choices. What other food system influences and stakeholders can also be addressed in order to create healthier food environments and support these rural communities?	The conclusion was improved as suggested. It was edited in the abstract and in the final conclusion of the paper
How was random selection done? Were all mothers with a child 6-23 months from all the villages enumerated and listed? Were pregnant women also included (if they also had a child 6-23 months)?	All women with children of age 6 to 23 months were listed in the village registry. The selection of the participants involved the use of random numbers. Those known to be pregnant were excluded in the study
Sample was 518 mothers 6 did not complete the dietary intake questionnaire then 6 were excluded during analysis due to special day of eating But sample reported in Table 1 is N=512, which does not compute accurately.	The reason for not completing the dietary intake questionnaire was that it was a previous day was a special day for them so this is the same 6 women. They were

	excluded in all the analysis hence remained with 512 throughout.
Does employed in agriculture mean that they are farming family or do they also work outside their own family farm on agriculture?	This means they also do farming. We have revised the term to read 'agriculture'
Is this a missing value? It is not reasonable for a household to have 0 monthly income, is it?	We kept it as a missing value as suggested
Table 4: To include bivariate analysis	Table 4a was added to represent a summary of NCD risk and protect
Similar to the NCD-Protect and NCD-Risk mean score, it would be nice to see this table include the mean DDS (dietary diversity score) for each of the dependent variables included in the model. You could have 4 columns in the table - the Variables, n, mean DDS (SD) and % with DDS >=5 (MDD-W) - Table 6a.	The table was changed to reflect the suggestion given
Reviewer 2	
Line 29: prevalence of overweight or obesity: overweight and obesity are not the same as they have different BMI cut-offs, so I would suggest rephrasing it such that they are complement (i.e. AND , not OR).	Revised as suggested (Abstract)
Line 30-31 the sentence on limited studies on diet quality: the mention of diet quality here is quite abrupt without any earlier indication on how diet is/might be related to malnutrition/overweight.	A sentence was added in the abstract to improve the connectivity of the statements.
Results: the statistics on education and owning mobile phone are not results of the study, rather they are descriptive statistics of the sample.<br><br>Vegetables and fried food consumption need to be defined, for example, did 70% consumed vegetables everyday? Does that imply that the remaining 30% do not consume vegetables at all?	The table title was improved to capture education and ownership of mobile phones.<br><br>The diet quality questionnaire asks for consumption in the last 24 hours as described in the methodology. The statement is now improved
Line 43: NCD-risk and protect score needs a brief explanation.	Explanation was included in the methodology section.
Line 44-45 mentions a number of factors that are associated with NCD-protect and risk scores. Given that one is a protection score and the other a risk score, the same household factors cannot be associated with both, without mentioning the direction of the association.	Corrected as suggested to show the direction
Introduction: Line 55: remove "a" before "low birth weight". Line 62: does it imply that undernutrition does not at all affect those who are not in the mentioned group at all?	Corrected as suggested. The sentence on undernutrition was qualified

Line 66-67: sentence unclear/check grammar.	Corrected
Line 73-74 needs reference.	Reference number 6 was cited.
Line 78: by [12]- incorrect method of citation. Similarly in line 247, 278, and others. Line 80: what is the age range for women of reproductive age?	All corrected as indicated in the revised document
Description of study area: this section can be made concise, e.g. the information on latitude, longitude and rainfall and irrigation schemes are somewhat unnecessary. More importantly, is the district primarily urban or rural?	Corrected as suggested
Line 98: unclear/confusing sentence, consider r	
The FoodLAND project is introduced abruptly without description or future mention of it. This section is 518 mothers while the abstract says 512.	Edited to remove the FoodLAND concept Total number of women sampled were 518 but analysis was included 512 who had complete dietary intake data
Line 147: "a single model with two dependent variables…" is misleading, kindly rephrase. I would assume that owning a mobile phone is correlated with household income, and if this is true, there is a possibility of collinearity between them. Similarly, income might be correlated to the household head being a male, explaining the results later that both income and having a male household head are associated with higher NCD-protect and risk scores and dietary diversity. I would suggest examining possible correlations between your dependent variables and if there is indeed a strong one, collinearity needs to be accounted for in the regression.	Some edits done on the model. The analysis was multivariate meaning that if income was a confounder, then it was controlled for during analysis
The variable names in equation 1 and 2 need to be defined.	The variables were defined
Results: the association between the dependent variables and the sociodemographic variables needs to be unpacked and explained (direction and strength of the relationship and what they actually mean).	The explanation of the results were improved. The meaning and interpretations were explained in the discussion section
Line 250: check grammar. Line 256-260: these two sentences are difficult to understand.	The two sentences were revised
Line 279-282: I would avoid making a comparison between findings from rural Tanzania and that from the US and UK, as they have a completely different demography, level of development, and food market.	Agreed with the suggestion. The sentence was edited
Some areas mention "NCD-protect" and sometimes "NCD-protective" is used.	Harmonized and used NCD-protect throughout the document
Line 349: male household heads may also have more resources, not just access? Refer to my earlier comment on possible correlation between male household head and	Reference number 1 and 44

household income. Is there any literature that shows male household heads is associated with better nutrition in the family?<br>Line 369-370: see earlier comment in abstract.	
Please review your reference list to ensure that it is complete and correct. If you have cited papers that have been retracted, please include the rationale for doing so in the manuscript text, or remove these references and replace them with relevant current references. Any changes to the reference list should be mentioned in the rebuttal letter that accompanies your revised manuscript. If you need to cite a retracted article, indicate the article's retracted status in the References list and also include a citation and full reference for the retraction notice.	Reference No 29 and 30 were replaced to include current studies conducted in similar settings<br>29. French SA, Tangney CC, Crane MM, Wang Y, Appelhans BM. Nutrition quality of food purchases varies by household income: The Shopper study. BMC Public Health. 2019;19[1]:1–7. **Replaced with**<br>Rashid, F.N., Sesabo, J.K., Lihawa, R.M. and Mkuna, E., 2024. Determinants of Household Food Expenditure in Tanzania: Implications on Food Security. Agriculture and Food Security, 13 (1), 1–16.<br>30. Pechey R, Monsivais P. Socioeconomic inequalities in the healthiness of food choices : Exploring the contributions of food expenditures. Preventive Medicine [Baltim] [Internet]. 2016;88:203–9. Available from: http://dx.doi.org/10.1016/j.ypmed.2016.04.012 **replaced with**<br>Headey, D.D., Ecker, O., Comstock, A.R. and Ruel, M.T., 2023. Poverty, price and preference barriers to improving diets in sub-Saharan Africa. *Global Food Security*, *36*, p.100664
**Comments from the Editor**	
Line 37: "A structure questionnaire was used to collect demographic data and the Global Diet Quality Questionnaire was used to collect dietary intake dat for women" instead of	We appreciate the comment. The sentence was revised as suggested

"A structured questionnaire and the Global Diet Quality Questionnaire for women were used to collect demographic and dietary intake data"	
Line 47: "employment or self-employment in agriculture" not "agriculture"	It was changed to self employment in agriculture
Line 52: suggest you define non-communicable diseases as "NCDs" not "NCD" and then use this consistently throughout	The sentence was changed as suggested and the abbreviation NCDs used consistently except in the NCD-protect and NCD-risk
Line 52: Capitalize Agriculture (Ministry of Agriculture)	The changes were effected in the respective line and in all other sections
Line 64: retain word "both"	The sentence was edited as suggested
Line 75: suggest "and of underweight is 10%" not "and underweight of 10%"	The sentence was corrected as suggested
Line 84: suggest "fat, salt" not "fat salt"	The sentence was changed. It now reads 'fat, sugar and salt.
Line 94: use acronym "NCDs" since you have defined it above (and for any subsequent references)	Changes were effected in the specific sentence and in other sections
Line 128: suggest "minimum dietary diversity score for women (MDD-W)" not "minimum dietary diversity score" (since MDD usually refers to the score for children). Then do a search and replace throughout to consistently abbreviate minimum dietary diversity score to MDD-W thereafter.	We acknowledge the mixing up of the dietary diversity indicators. Minimum dietary diversity for women (MDD-W) is now used throughout the document
Line 130 "are free" not "it is free" (tools is plural)	The sentence was changed
Line 133: suggest you are consistent in the titles of the scores: e.g. "NCD-protect" and "NCD-risk" throughout (or "NCD-Protect" and "NCD-Risk" as in lines 134 and 140) but don't switch between them. Do a search and replace. Also, keep them in the same order throughout when talking about both scores e.g. NCD-protect and risk, don't switch to NCD-risk and protect as occurs at subsequent points in the text.	We accept the suggestion and we have effected the proposed changes throughout the document.
Line 154: "were used" not "was used" (variables, used in line 153, is plural)	Changed as proposed
Line 156 "Econometric model" not "Econometrics model"	Changed as proposed
Line 161: "were analyzed" (models is plural)	Changed
Line 171: delete "monthly" from food expenditure – if the expenditure is all monthly then define it that way at first usage, and thereafter do not add "monthly". Need to be consistent	The suggestion was effected throughout the document
Lines 174 and 186: suggest "Agricultural Employment/Self-employment" – if defined here, then you can use "agricultural" as a short form later	Defined as agricultural employment and retained agricultural throughout the document
Lines 175 and 187: "Sex of head of household is Male" (if that is correct)	Yes, changed accordingly

Line 188: suggest you use title Minimum Dietary Diversity for Women; and you don't need to redefine it in line 189 and 193 as it has been defined above.	The abbreviation MDD-W is now used throughout the document and the definition was deleted
Line 91: suggest add ":" before commencing the list	Changed as suggested
Line 193: suggest end the list with "and other vegetables and fruit	Changed as suggested
Line 199: I think you mean "socio-demographic" not "social demographic"	The sentence was changed to read socio-demographic (pg 6)
Line 219: if you have redefined 0 monthly income as missing, then you need to give the actual minimum in line 219 and in the table below. Also in line 219, replace "1,550,000" with "1,550,000 TZS" for clarity, and state "mean monthly household income" not "average household income"	Minimum income level was changed to 3000TZS and TZS was added on 1,550,000 as suggested
Line 213: suggest "26.4, with a standard deviation of 7.3" not "26.4 +/- 7.3" – this isn't a confidence interval.	Changed as suggested
Line 229 and Table 3: use term baked/ grain-based sweets consistently (same format as QDQQ), here and elsewhere.	Changed in all sections as suggested
Line 241: Table 4b: add in table header NCD-Protect (mean+/- SD) and NCD-Risk (mean +/- SD). Also integrate table 4a as 2 additional lines in table 4b. You don't need to retain the data on the ranges, since you don't have those in table 4b.	Header was changed and the two tables were combined as suggested
Table 5: either state "Agricultural" or "Agricultural employment/self-employment" to be consistent. Also reorganize lines 153 and 154 as 4 additional lines of Table 5 (one row for RMSE, R-square and F. You can add asterisks to indicate significance to the F statistics, and then no row for P is required.	Changes as suggested
Table 6: make title Minimum Dietary Diversity for Women Score and replace DDS by MDD-W within the table	Changed as proposed
Renumber Table 6b as Table 7 (and change text references consistently). Consider carefully whether you really need 4 decimal places throughout table. 2 will likely suffice. Also relabel "Agricultural employment" either as "Agricultural" or "Agricultural employment/self-employment"	Tables were re-numbered and decimal places reduced to 2. Other changes were effected as suggested
Line 321: "younger counterparts" not "counterparts"	Changed as recommended
Line 345: "NCD-risk" not "NCDs-risk"	The letter 's' was deleted
Line 350: "french fries" not "French fries"	Changed as suggested
Line 355: "minimum" not "medium	Changed as suggested
Line 387: "chores which" not "chores"	Changed as suggested
Line 390: "MDD-W" not "minimum dietary diversity"; note that this change should be made throughout. MDD refers to children's diet diversity, and data on this has not been presented in this study	Apologies for the incorrect wording. We changed all the sections as suggested
In line 398, do these other studies look at "adequate dietary diversity" or do they look at MDD-W?	Yes, the two studies looked at the MDD-W not the adequate dietary diversity

Line 407: “MDD-W” not “MDD) – same as previous comment.	Changed as suggested
Line 411: Ministry of Agriculture” not “Ministry of agriculture”; similar comment re “ministry of education” in following sentence.	Changed as suggested. Both Ministry of Agriculture and Ministry of Education are now written correctly
