# Supplementary material for: Consumption of protect and risk food groups for non-communicable diseases among women of reproductive age in rural areas of Morogoro, Tanzania
Source: PLoS One. 2025 Feb 25;20(2):e0318744. doi: 10.1371/journal.pone.0318744 (PMC11856286; doi:10.1371/journal.pone.0318744)
Supplement: Attachment — Submitted filename: Response to reviewers_Plos ONE_30 Sept 2024_AWM.docx [file pone.0318744.s002.docx]

**Response to reviewers’ comments: Oct 2024**

**Dear Editor,**

We appreciate the comments and suggestions given by the reviewers.

We hereby report the revisions made and submit for your considerations

| **Reviewers comments** | **Response** |
| --- | --- |
| Please state what role the funders took in the study. If the funders had no role, please state: "The funders had no role in study design, data collection and analysis, decision to publish, or preparation of the manuscript."  If this statement is not correct you must amend it as needed.  Please include this amended Role of Funder statement in your cover letter; we will change the online submission form on your behalf. | The role of the funders is stated in the submission letter. It is also included in source of funds section in the manuscript |
| When completing the data availability statement of the submission form, you indicated that you will make your data available on acceptance. We strongly recommend all authors decide on a data sharing plan before acceptance, as the process can be lengthy and hold up publication timelines. Please note that, though access restrictions are acceptable now, your entire data will need to be made freely accessible if your manuscript is accepted for publication. This policy applies to all data except where public deposition would breach compliance with the protocol approved by your research ethics board. If you are unable to adhere to our open data policy, please kindly revise your statement to explain your reasoning and we will seek the editor's input on an exemption. Please be assured that, once you have provided your new statement, the assessment of your exemption will not hold up the peer review process. | The data will be made available. The project consortium is aware of the requirement and ready to share data when needed |
| Please amend either the abstract on the online submission form (via Edit Submission) or the abstract in the manuscript so that they are identical. | Amendment of the abstract was done to make the one online identical with the manuscript |
| Your ethics statement should only appear in the Methods section of your manuscript. If your ethics statement is written in any section besides the Methods, please move it to the Methods section and delete it from any other section. Please ensure that your ethics statement is included in your manuscript, as the ethics statement entered into the online submission form will not be published alongside your manuscript. | The ethics statement in the abstract was deleted |
| The results are largely consistent with the nationally representative survey conducted in 2021 by Gallup Poll of 1000 women and men in both urban and rural areas of Tanzania, although the authors of this study do not seem to be aware of that data. | We appreciate the comment on the additional manuscript published in 2021. We included it to improve our discussion |
| What time of year the data were collected (especially with respect to seasonal food availability),  how random sampling of the women across the villages was carried out, and  who conducted the data collection (number of people, training provided, any assessment of inter-rater reliability).  With respect to the results, it would be helpful to have a full description of the MDD-W results by dependent variable (Table 6a) - see my suggestions in the attached PDF file. Furthermore, the results of the logistic regression model used to explore the association of the dependent variables with MDD-W would be easier to interpret if the coefficients were presented as odds ratios with 95% confidence intervals | The mothers were selected randomly from the village registry which had a list of all women with children of age 6 to 23 months  10 enumerators (all females) were trained to collect data. The data collection tool was pretested and checking for consistency in asking questions.  Table 6 on the MDD-W was improved to include OR and CI as suggested (Table 6b); Pg 16 |
| I noted that the minimum dietary diversity indicator is frequently incorrectly described as 'medium dietary diversity' throughout the manuscript. Also, there were several instances where the in-text citation of a study was done incorrectly. Please add the name of the study author when referring to an article in the text rather than saying "a study done by [19]" - example from line 247. | Thank you for the observation. We admit this incorrect way of citation. It was corrected throughout the manuscript |
| In both the results and discussion sections, I found that it would be helpful to consider these women more holistically, looking at patterns or groups within the dataset. For example, rather than looking at independent associations of age and mobile phone ownership, were there patterns across these characteristics? Were the women who owned mobile phones also more likely to be younger women? Similarly for the discussion of NCD-protect and MDD-W, there is a large amount of overlap in these two metrics of diet quality. While they are distinct indicators designed to be interpreted in different ways, there did not seem to be a recognition that the same dietary intake patterns that drive one also drive the other. Rather than discussing them separately, it would be helpful to consider what are the common underlying factors that make it difficult for women to consume more fruits and vegetables, for example.  Finally, I found the discussion of implications of these results a bit sparse, limited to one sentence in the Conclusion section. Rather than placing all the responsibility on these women and their communities for increased production, increased incomes and better food choices, what are the potential implications for agriculture, food and healthy diet policies in the country? Given that households may start to purchase more food as incomes increase, what are the options for ensuring that more diverse and healthy foods are available for purchase in this district, even if it is not possible for the communities to produce all of it themselves? What role can public health actors play in providing nutrition education to all ages, including young people, to enhance awareness of foods that are associated with increased risk of NCDs? And given the challenge in meeting nutrient adequacy with locally available foods, how can the country's food fortification agenda be scaled up to benefit women in these communities? These are some possible questions for the authors to consider as they expand a bit more on what can be done in response to these findings. | It is true that the women who owned mobile phones were younger. Generally, 50% of those of age 15 to 25 had mobile phones compared to 36% among 26 to 35 years and 14% for those older than 35 years  However, all this variables were entered in the model together to control for confounding effect. The discussion section was revised as suggested  We appreciate the comment. The conclusion was revised as suggested |
| The conclusion here appears to put the onus on rural communities to increase their knowledge (education) about diet diversification, increased income and healthy food choices. What other food system influences and stakeholders can also be addressed in order to create healthier food environments and support these rural communities? | The conclusion was improved as suggested. It was edited in the abstract and in the final conclusion of the paper |
| how was random selection done? Were all mothers with a child 6-23 months from all the villages enumerated and listed? Were pregnant women also included (if they also had a child 6-23 months)? | All women with children of age 6 to 23 months were listed in the village registry. The selection of the participants involved the use of random numbers. Those known to be pregnant were excluded in the study |
| Sample was 518 mothers 6 did not complete the dietary intake questionnaire then 6 were excluded during analysis due to special day of eating But sample reported in Table 1 is N=512, which does not compute accurately. | The reason for not completing the dietary intake questionnaire was that it was a previous day was a special day for them so this is the same 6 women. They were excluded in all the analysis hence remained with 512 throughout. |
| Does employed in agriculture mean that they are farming family or do they also work outside their own family farm on agriculture? | This means they also do farming. We have revised the term to read ‘agriculture’ |
| Is this a missing value? It is not reasonable for a household to have 0 monthly income, is it? | We kept it as a missing value as suggested |
| Table 4: To include bivariate analysis | Table 4a was added to represent a summary of NCD risk and protect |
| Similar to the NCD-Protect and NCD-Risk mean score, it would be nice to see this table include the mean DDS (dietary diversity score) for each of the dependent variables included in the model. You could have 4 columns in the table - the Variables, n, mean DDS (SD) and % with DDS >=5 (MDD-W) - Table 6a. | The table was changed to reflect the suggestion given |
|  |  |
| Reviewer 2 |  |
| Line 29: prevalence of overweight or obesity: overweight and obesity are not the same as they have different BMI cut-offs, so I would suggest rephrasing it such that they are complement (i.e. AND , not OR). | Revised as suggested (Abstract) |
| Line 30-31 the sentence on limited studies on diet quality: the mention of diet quality here is quite abrupt without any earlier indication on how diet is/might be related to malnutrition/overweight. | A sentence was added in the abstract to improve the connectivity of the statements. |
| Results: the statistics on education and owning mobile phone are not results of the study, rather they are descriptive statistics of the sample.  Vegetables and fried food consumption need to be defined, for example, did 70% consumed vegetables everyday? Does that imply that the remaining 30% do not consume vegetables at all? | The table title was improved to capture education and ownership of mobile phones.  The diet quality questionnaire asks for consumption in the last 24 hours as described in the methodology. The statement is now improved |
| Line 43: NCD-risk and protect score needs a brief explanation. | Explanation was included in the methodology section. |
| Line 44-45 mentions a number of factors that are associated with NCD-protect and risk scores. Given that one is a protection score and the other a risk score, the same household factors cannot be associated with both, without mentioning the direction of the association. | Corrected as suggested to show the direction |
| Introduction: Line 55: remove “a” before “low birth weight”. Line 62: does it imply that undernutrition does not at all affect those who are not in the mentioned group at all? | Corrected as suggested. The sentence on undernutrition was qualified |
| Line 66-67: sentence unclear/check grammar. | Corrected |
| Line 73-74 needs reference. | Reference number 6 was cited. |
| Line 78: by [12]- incorrect method of citation. Similarly in line 247, 278, and others. Line 80: what is the age range for women of reproductive age? | All corrected as indicated in the revised document |
| Description of study area: this section can be made concise, e.g. the information on latitude, longitude and rainfall and irrigation schemes are somewhat unnecessary. More importantly, is the district primarily urban or rural? | Corrected as suggested |
| Line 98: unclear/confusing sentence, consider r |  |
| the FoodLAND project is introduced abruptly without description or future mention of it.  This section is 518 mothers while the abstract says 512. | Edited to remove the FoodLAND concept  Total number of women sampled were 518 but analysis was included 512 who had complete dietary intake data |
| Line 147: “a single model with two dependent variables…” is misleading, kindly rephrase. I would assume that owning a mobile phone is correlated with household income, and if this is true, there is a possibility of collinearity between them. Similarly, income might be correlated to the household head being a male, explaining the results later that both income and having a male household head are associated with higher NCD-protect and risk scores and dietary diversity. I would suggest examining possible correlations between your dependent variables and if there is indeed a strong one, collinearity needs to be accounted for in the regression. | Some edits done on the model.  The analysis was multivariate meaning that if income was a confounder, then it was controlled for during analysis |
| The variable names in equation 1 and 2 need to be defined. | The variables were defined |
| Results: the association between the dependent variables and the sociodemographic variables needs to be unpacked and explained (direction and strength of the relationship and what they actually mean). |  |
| Line 250: check grammar. Line 256-260: these two sentences are difficult to understand. | The two sentences were revised |
| Line 279-282: I would avoid making a comparison between findings from rural Tanzania and that from the US and UK, as they have a completely different demography, level of development, and food market. | Agreed with the suggestion. The sentence was edited |
| Some areas mention “NCD-protect” and sometimes “NCD-protective” is used. | Harmonized and used NCD-protect throughout the document |
| Line 349: male household heads may also have more resources, not just access? Refer to my earlier comment on possible correlation between male household head and household income. Is there any literature that shows male household heads is associated with better nutrition in the family? Line 369-370: see earlier comment in abstract. | Reference number 1 and 44 |
|  |  |
